# Responses to a warming world: Integrating life history, immune investment, and pathogen resistance in a model insect species

**DOI:** 10.1002/ece3.3506

**Published:** 2017-10-16

**Authors:** Alice M. Laughton, Cian O. O'Connor, Robert J. Knell

**Affiliations:** ^1^ School of Biological and Chemical Sciences Queen Mary University of London London UK

**Keywords:** defense, ecological immunology, global warming, hemocyte, phenoloxidase, *Plodia interpunctella*, trade‐off

## Abstract

Environmental temperature has important effects on the physiology and life history of ectothermic animals, including investment in the immune system and the infectious capacity of pathogens. Numerous studies have examined individual components of these complex systems, but little is known about how they integrate when animals are exposed to different temperatures. Here, we use the Indian meal moth (*Plodia interpunctella*) to understand how immune investment and disease resistance react and potentially trade‐off with other life‐history traits. We recorded life‐history (development time, survival, fecundity, and body size) and immunity (hemocyte counts, phenoloxidase activity) measures and tested resistance to bacterial (*E. coli*) and viral (*Plodia interpunctella* granulosis virus) infection at five temperatures (20–30°C). While development time, lifespan, and size decreased with temperature as expected, moths exhibited different reproductive strategies in response to small changes in temperature. At cooler temperatures, oviposition rates were low but tended to increase toward the end of life, whereas warmer temperatures promoted initially high oviposition rates that rapidly declined after the first few days of adult life. Although warmer temperatures were associated with strong investment in early reproduction, there was no evidence of an associated trade‐off with immune investment. Phenoloxidase activity increased most at cooler temperatures before plateauing, while hemocyte counts increased linearly with temperature. Resistance to bacterial challenge displayed a complex pattern, whereas survival after a viral challenge increased with rearing temperature. These results demonstrate that different immune system components and different pathogens can respond in distinct ways to changes in temperature. Overall, these data highlight the scope for significant changes in immunity, disease resistance, and host–parasite population dynamics to arise from small, biologically relevant changes to environmental temperature. In light of global warming, understanding these complex interactions is vital for predicting the potential impact of insect disease vectors and crop pests on public health and food security.

## 1 INTRODUCTION

Temperature is the single most important abiotic factor influencing the biology of ectothermic animals. Dependence on the external environment for body temperature control has significant impacts on insect physiological functioning (Chown & Nicolson, 2004) and, subsequently, life‐history traits (Ciota, Matacchiero, Kilpatrick, & Kramer, 2014; Clissold & Simpson, 2015; Lachenicht, Clusella‐Trullas, Boardman, Le Roux, & Terblanche, 2010). Temperature effects may be particularly important when considering the immune system, as responses to temperature variation can have direct consequences for both pathogen infection and host survival (Alto & Bettinardi, 2013; Murdock, Blanford, Luckhart, & Thomas, 2014; Pamminger, Steier, & Tragust, 2016; Pounds et al., 2006; Richards, Anderson, Lord, & Tabachnick, 2012; Wolinska & King, 2009).

The immune system of insects comprises constitutive and induced responses (Schmid‐Hempel, 2005). Induced components are pathogen‐specific and take longer to produce, whereas constitutive immune responses are nonspecific and immediate in their action. Constitutive responses include the cellular actions of coagulation, encapsulation, and phagocytosis, which require the recruitment of hemocytes following recognition of an immune challenge (Hillyer, 2016), and the phenoloxidase activation system (PO‐AS). Active phenoloxidase (PO) is required for initiating the production of melanin (Gillespie, Kanost, & Trenczek, 1997), which has multiple functions in immunity, being used for cuticle sclerotization, clotting, and encapsulation responses (Cerenius, Lee, & Söderhäll, 2008; Cerenius & Söderhäll, 2004).

Understanding how temperature affects the disparate components of the immune system and interactions therein is important for predicting the phenotypic outcome of the organism. While increasing temperature tends to increase the rate of biochemical processes, this does not necessarily extend to all aspects of physiology, and increasing temperature may not positively correlate with improved immune competence (Angilletta, Huey, & Frazier, 2010; Murdock et al., 2012; Suwanchaichinda & Paskewitz, 1998).

Rates of enzymatic‐based immune responses, such as PO activation, have been shown to increase positively with increasing temperature within a threshold range (Adamo & Lovett, 2011; Catalán, Niemeyer, & Kalergis, 2012; Ferguson, Heinrichs, & Sinclair, 2016; Fuller, Postava‐Davignon, West, & Rosengaus, 2011), but other commonly recorded immune measures report a more moderate optimum. The ability of mosquitoes to melanize sephadex beads, for example, significantly decreased as temperature increased from 24–30°C (Suwanchaichinda & Paskewitz, 1998) and has been shown to peak at 18°C (Murdock et al., 2012). Consequently, the relative efficiency of different immune responses is likely to vary with temperature (Murdock et al., 2012), potentially resulting in trade‐offs at different optima (Cotter, Myatt, Benskin, & Wilson, 2008; Freitak, Wheat, Heckel, & Vogel, 2007).

Maintaining an immune system is costly (Rolff & Siva‐Jothy, 2003; Schmid‐Hempel, 2005), and while interrelated immune parameters may impact each other, they are also in resource competition with other life‐history traits. Trade‐offs have been reported between immune measures and development rate, reproductive activity, and fecundity (see Schmid‐Hempel, 2005; Schwenke, Lazzaro, & Wolfner, 2015; for reviews): all of which could be further influenced by the effects of temperature. These species‐specific trade‐offs are further compounded by the introduction of temperature as an external ecological stressor. For example, a short exposure to temperature stress in the butterfly *Bicyclus anynana* produced contrary responses between life‐history and immune measures that became more pronounced when individuals were nutritionally depleted, indicating trade‐offs in resource allocation (Karl, Stoks, De Block, Janowitz, & Fischer, 2011).

The ability to predict genotype‐by‐environment (GxE) interactions in determining host fitness becomes even more complex if host–pathogen interactions are considered (GxGxE). Pathogen exposure can also exacerbate preexisting trade‐offs between the immune system and other life‐history measures (Schwenke et al., 2015). Consequently, a combination of temperature and pathogen stressors acting on these interactions may provoke a more striking outcome.

Environmental temperature can have a significant impact on host–pathogen interactions, as it affects both immune system functioning, and consequently a host's ability to resist or tolerate infections, and pathogen virulence and population dynamics. The strongly nonlinear response of these factors to temperature changes makes predicting the outcomes of infection particularly challenging (Sternberg & Thomas, 2014). Relatively small and realistic changes in temperature have a significant effect on the virulence of a wide range of microbial pathogens in invertebrate hosts (see Thomas & Blanford, 2003 for review), leading to potential conflicts over optimal operating temperatures for both parties. For example, the faster growth rate of *Aspergillus* fungus in the sea fan coral, *Gorgonia ventalina*, at higher temperatures is mirrored by an increase in the activity of host‐derived antifungal compounds (Ward, Kim, & Harvell, 2007). However, temperature‐associated increases in pathogen replication rates may be so rapid as to overwhelm the capacity of the host immune system. Conversely, increasing temperature may reduce host lifespan, so perturbing pathogen transmission. Alternatively, hosts may be able to use temperature to their advantage, employing behavioral thermoregulation to induce a fever that restricts pathogen growth (Elliot, Blanford, & Thomas, 2002). Work has started to investigate the temperature effects on immune responses and life‐history parameters following challenge with an immune elicitor (Catalán et al., 2012). However, temperature stress was only applied postchallenge. In mosquitoes, environmental temperature experienced during development was found to have significant effects on resistance to dengue virus (Alto & Bettinardi, 2013), highlighting the importance of host lifetime thermal experience in understanding these interactions.

The Indian meal moth, *Plodia interpunctella*, is a global pest responsible for damage to stored grains and cereal products. Being able to survive and reproduce within a relatively broad temperature range (~16–35°C) makes *Plodia* a serious problem in tropical, subtropical, and temperate regions (Arbogast, 2007; Johnson, Wofford, & Whitehand, 1992; Mohandass, Arthur, Zhu, & Throne, 2007; Na & Ryoo, 2000; Tzanakakis, 1959). The moths are, however, highly sensitive to changing ecological factors (Boots & Roberts, 2012; Johnson et al., 1992; Littlefair, Laughton, & Knell, 2017; Triggs & Knell, 2012), including temperature (Triggs & Knell, 2012). While developmental rates increase positively with temperature (Arbogast, 2007; Na & Ryoo, 2000), adult longevity, and fecundity declines, with a fecundity optimum occurring around a moderate 25°C (Arbogast, 2007). Recent work has shown that temperature effects on *Plodia* immune parameters are strongly dependent on other environmental factors, indicating complex trade‐offs within the system (Triggs & Knell, 2012). Given its propensity for global proliferation, much *Plodia* research has focused on the development of biopesticides as control strategies (e.g., Giles, Hellmich, Iverson, & Lewis, 2000; Oluwafemi, Rao, Wang, & Zhang, 2009). However, to date, no work has linked the relationship between environmental temperature, effects on life‐history and immune measures, and subsequent outcomes for host–pathogen interactions.

Here, we use a biologically relevant temperature range (20–30°C) to assess how life‐history traits (developmental time, body size, longevity, and fecundity) and immune measures (PO activity and hemocyte counts) vary in response to relatively small temperature increments. PO activity and hemocyte counts are commonly measured to establish immune capacity in insects (Schmid‐Hempel, 2005), both being associated with resistance to a range of pathogens and parasites (e.g., Reeson, Wilson, Gunn, Hails, & Goulson, 1998; Prevost & Eslin, 1998; Contreras‐Garduño, Lanz‐Mendoza, & Córdoba‐Aguilar, 2007; Pauwels, De Meester, Decaestecker, & Stoks, 2010; Valadez‐Lira et al., 2012; Poyet et al., 2013; but see González‐Santoyo & Córdoba‐Aguilar, 2012; for review). Finally, using naturally occurring pathogens (*E. coli* and *Plodia interpunctella* granulosis virus [PiGV]), we compare the impact of temperature on the ability of *Plodia* to respond to septic injury and resist infection.

## 2 METHODS

### 2.1 Study animal

An outbred *Plodia interpunctella* stock culture was established in 2013 by combining three stock lines (one maintained at the University of Liverpool for at least a decade, one collected in Perth, Australia in 2001, and a commercially available line maintained at Fera Science Limited). Moths were reared in a 12L:12D light cycle at 26°C, and larvae were fed on a standard diet of 10:1:1 ratio of organic wheat bran (Mount Pleasant Mill, Lincolnshire), brewer's yeast, and glycerol. The outbred line was allowed to establish for at least 10 generations before being used for experiments.

### 2.2 Temperature treatments

The effects of temperature were measured at 20, 22, 24, 27, and 30°C, with animals for each treatment group being maintained in separate incubators set at the appropriate temperature (±0.5°C), the incubators themselves being kept in a controlled temperature room set at 20°C. Humidity was not controlled but is generally low in this facility.

### 2.3 Life‐history measures

To determine the effect of a temperature gradient on life‐history measures, eggs collected from the stock population over a 24‐hr period were divided equally between containers in the five incubators with ad lib food. Larvae were allowed to hatch and grow to fourth instar (in all cases, developmental stage is determined by head capsule size, with the ratio of head capsule to larval body judged by eye to control for body size), then sexed (testicles are easily identified through the cuticle), and 60 females per temperature treatment put into individual Petri dishes with 1 g of food. The subsequent time to pupation and adult eclosion was recorded. On eclosion, 30 randomly chosen females from each temperature group were paired with virgin males retained from the same treatment group and allowed to mate for 24 hr, after which time the males were removed and discarded. Mated females were transferred to a fresh pot every 48 hr, and eggs laid in the previous 48 hr were counted. This was continued until female death. Nonmated females were also maintained and time to death recorded.

To measure the effect of temperature on body size, eggs were set up as before, and animals allowed to develop to adult emergence. The wing lengths of twenty randomly selected females from each treatment group were then measured (using ImageJ software) as a proxy for body size.

### 2.4 Immune measures

As above, stock eggs were split between five incubators at 20, 22, 24, 27, and 30°C and allowed to develop to fifth‐instar larvae with ad lib food. Due to variation in the rate of development between the temperature treatment groups, samples were collected over a period of 17 days. Larval weight was recorded to control for variation in body size between the temperature treatments. Larvae were sexed, and the females selected for use in either hemocyte count and phenoloxidase (PO) sample collection or bacterial clearance assays. Collecting samples for the hemocyte count and PO assays from the same individual allowed for direct comparison of the influence of temperature on both immune assays in individual larvae.

Due to collection time constraints, assay samples were collected in two blocks, resulting in final sample sizes of 37–64 per immune assay per temperature treatment.

#### 2.4.1 Hemocyte counts

Hemocytes counts, both total and relative, have been found to correlate with an increased immune function, with higher hemocyte counts being associated with a stronger encapsulation response (Kraaijeveld & Godfray, 2001; Poyet et al., 2013; Prevost & Eslin, 1998; Wilson, Knell, Boots, & Koch‐Osborne, 2003). Two microliters of hemolymph was collected from each fifth‐instar larva. One microliter of this was immediately frozen at −80°C for use in determining PO activity. The remaining 1 μl was thoroughly mixed with 7 μl anticoagulant buffer (4:3 glycerol:EDTA anticoagulant [EDTA 2.9225 g + citric acid 1.9213 g + 80 ml PBS, pH 7.4]), and frozen (−80°C) for hemocyte counting. All cell counts were carried out within 3 weeks of collection to avoid the hemocyte deterioration seen in older frozen samples (A. M. Laughton, pers. obs.). To count, samples were defrosted on ice, mixed again by pipette to ensure even cell distribution, and 6 μl added to a hemocytometer (Neubauer chamber, 0.1 mm depth). Total hemocyte counts were recorded from five predetermined cells within the hemocytometer grid and averaged to give a total per sample (*n* = 37–43 per temperature treatment).

*Plodia* have six different types of hemocytes, some of which have functions in the immune system (Beeman, Wilson, Bulla, & Consigli, 1983; Ribeiro & Brehélin, 2006). These are only distinguishable in fresh preparations or using electron microscopy; however, and because we used frozen material with an anticoagulant buffer, we were unable to reliably separate cell types. Consequently, only total hemocyte counts (THC) were recorded, but we note that this approach has worked well in previous studies and THC is the measure that has been shown consistently to correlate with encapsulation (e.g., Eslin & Prevost, 1996; Kraaijeveld & Godfray, 2001; Wilson et al., 2003).

#### 2.4.2 Phenoloxidase activity

Phenoloxidase (PO) is present in the hemolymph as both the active enzyme and as inactive proPO. Most studies of insect immune reactivity focus on the active enzyme, although some have studied both total PO, with the proPO activated using chymotrypsin. Some of these have found that levels of active and total PO are very strongly correlated (e.g., Körner, Vogelweith, Foitzik, & Meunier, 2017), whereas others have found that the two measures respond differently to other aspects of the animal's biology (e.g., Busso, Blanckenhorn, & González‐Tokman, 2017). Here, for logistic reasons and to make our results comparable with the majority of other work, we report on active PO activity only. PO activity was assayed by measuring its catalytic conversion of L‐dopa (3,4‐dihydroxy‐L‐phenylalanine, colorless) to dopachrome (red‐brown color) photometrically (Barnes & Siva‐Jothy, 2000; Horowitz & Shen, 1952). One microliter of hemolymph samples was defrosted on ice, 9 μl cold phosphate‐buffered saline (PBS, Sigma) added, and the sample vortexed to mix. The 10 μl sample was added to the well of a chilled 96‐well plate. L‐dopa (100 μl, 4 mg/ml in dH_2_0) was added, and the reaction was allowed to proceed at 30°C in a spectrophotometer (Multiskan Ascent, Thermo Labsystems). Readings were taken every 15 s for 1 hr at 490 nm and analyzed using Ascent v 2.6 software. Enzyme activity (Vmax) was measured as the maximum linear rate of substrate conversion. Final sample size was *n* = 37–43 per temperature treatment.

#### 2.4.3 Bacterial clearance

Bacterial clearance assays in insects are widely performed using *Escherischia coli* to challenge the host's immune response (e.g., Castillo, Brown, & Strand, 2011; Elrod‐Erickson, Mishra, & Schneider, 2000). *E. coli* are mesophilic bacteria with a temperature growth range of 25–40°C and an optimal growth temperature of 37°C (Nguyen, 2006; Sutherland, Bayliss, & Braxton, 1995). Bacterial growth rate is expected to increase linearly with temperature within the experimental temperature range used here (Ratkowsky, Olley, McMeekin, & Ball, 1982).

To assess the effect of temperature on the ability of *Plodia* larvae to clear a bacterial infection, female fifth‐instar larvae were injected laterally with 8 μl tetracycline‐resistant *E. coli* (strain BC‐Gold, obtained from the Sullivan Lab [QMUL]) at OD_60_ = 1 using a Hamilton syringe. A couple of drops of red food dye were added to the standardized bacterial broth to enable monitoring of successful injections. Injected larvae were allowed to recover in a Petri dish for 10 min before being placed into a treatment pot with ad lib food and returned to the appropriate incubator. Twenty‐four hours postchallenge, surviving larvae from each treatment pot were collected, individually placed into 1.5‐ml microtubes and homogenized with 200 μl PBS using sterile pestles to ensure the capture of all remaining bacteria. Samples were further diluted to 1:1,000 μl PBS, vortexed, and 50 μl subsample was plated onto LB plates with tetracycline using a sterile spreader. Plates were incubated at 37°C for 24 hr, and any resulting colonies (colony‐forming units, CFUs) counted. Original samples were retained at 4°C for 24 hr if required for any necessary replating or further dilution. Retaining samples for this period was not found to significantly alter bacterial counts (A. M. Laughton, pers. obs.). Final sample sizes were *n* = 43–64 per temperature treatment.

### 2.5 Virus resistance

Virus resistance was measured using the naturally occurring virus *Plodia interpunctella* granulosis virus (PiGV). Following the oral ingestion of occlusion bodies by larvae, virus particles infect the midgut epithelial cells and spread into secondary tissues, leading to cell lysis, tissue damage, and, ultimately, host death prior to pupation (Begon, Daud, Young, & Howells, 1993). Infected larvae turn a characteristic opaque white color, so are easily distinguished from healthy individuals. PiGV resistance was assayed using an LD_50_ dose (predetermined from stock solution as 0.5 μl virus solution + 999.5 μl sucrose solution [50:50 sucrose:dH_2_O]). Stock virus solution was made by centrifuging a homogenate of infected individuals following Boots and Begon (1994). All virus applications were made using the same stock solution aliquoted into doses and stored at −20°C. Virus was administered orally to early third ‐instar larvae (mixed sex, 6–14 days old depending on rearing temperature, developmental stage determined by relative head capsule width to body size). Third‐instar larvae were used for this assay because final (fifth) instar *P. interpunctella* are completely resistant to PiGV and fourth‐instar larvae, while susceptible, require large doses to become infected (Sait, Begon, & Thompson, 1994). Final‐instar larvae, however, are much easier to handle because of their size and procedures such as injection or hemolymph extraction are considerably easier, and hence, they were used for the other experiments in this study. Larvae were removed from the diet medium and left to starve for 2 hr prior to dosing. Each larva was then presented with a 1 μl droplet of virus/sucrose solution. Red food dye was added to the sucrose solution, and larvae deemed to have been successfully dosed when dye could be seen filling at least half the length of their gut.

Due to the differences in larval growth rates between the temperature treatments, virus resistance was assayed in two ways. Eggs were collected from the stock line during a 24‐hr period, and all treatment groups were established by placing ~40 eggs onto 12 g food in a small pot. For the first assay method, treatment pots were then divided equally between the five temperature treatment incubators. Larvae were allowed to develop to third instar, at which point they were dosed with virus (as above). Virus administration was staggered over an 8‐day period to allow for each temperature treatment group to reach the same developmental stage, with larvae judged by eye to determine size for infection. Postinoculation, larvae were individually placed into 25‐well Petri dishes with *ad lib* food, returned to their respective incubators, and checked 2 weeks later for signs of infection.

In the second assay method, the larvae were all raised in treatment pots at 26°C (standard *Plodia* lab rearing conditions) until third instar, ensuring that larval development was synchronized. Larvae were inoculated with virus on the same day and after dosing were divided equally across the five temperature treatment incubators and allowed to continue development for 2 weeks (in 25‐well Petri dishes with ad lib food) before checking for signs of disease. Sample sizes postinoculation ranged from 25 to 50, although due to some larval mortality, final sample sizes 2 weeks postinoculation were reduced to *n* = 18–30.

### 2.6 Analysis

All data analyses and relevant R code are available in the supporting information, as are the data sets analyzed. All data were analyzed using R v3.2.3 (R Core Team, 2015). Linear mixed‐effects models were fitted to the majority of response variables, with temperature as a continuous explanatory variable and incubator as a random factor. Experimental block and larval weight were fitted as covariates when appropriate. When exploratory analysis and inspection of diagnostic plots indicated a curved relationship between temperature and a response variable, polynomial models were fitted. In a few cases, the response variable was transformed when diagnostic plots indicated deviation from model assumptions. Most models used normal errors, but virus infection was analyzed using binomial errors. The effects on survival were modeled using a mixed‐effects Cox proportional hazards model fitted using the coxme package (Therneau, 2015) with no censoring (data conformed to model assumptions).

The effect of temperature on egg production was analyzed using a generalized additive mixed‐effects model with quasi‐Poisson errors (GAMM), fitted using the mgcv package (Wood, 2006; Zuur, Ieno, Walker, Saveliev, & Smith, 2009). Individual moth ID and incubator were included as random factors and separate smoothers were fitted for each level of temperature.

Mixed‐effects models have to be used cautiously when the number of levels of a random factor is low (see, e.g., http://glmm.wikidot.com/faq). In this study, there were five incubators, a sufficiently small number to sometimes cause problems during model fitting. These problems usually manifest as failure to converge, unrealistically large coefficient estimates or estimates of zero for the variance component associated with a random factor, and during the model fitting process, we were alert to these potential issues. Only the model fitted to the hemocyte count data indicated any of these issues, returning an estimated variance of zero for the random factor. This model also displayed some positive skew in the distribution of residuals, and a square‐root transformation of the response variable corrected both of these problems.

## 3 RESULTS

### 3.1 Life‐history measures

**Figure 1:**
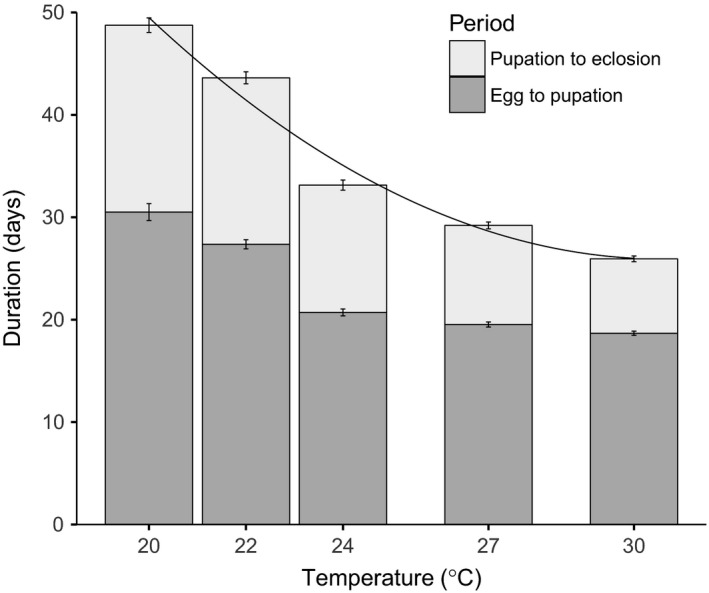
Temperature effects on *Plodia* developmental time showing both egg‐to‐pupa time and pupal duration. The full bar is therefore the egg‐to‐eclosion time. Error bars are ±1 *SEM*. The line shows the predicted values from the fitted quadratic model

**Figure 2:**
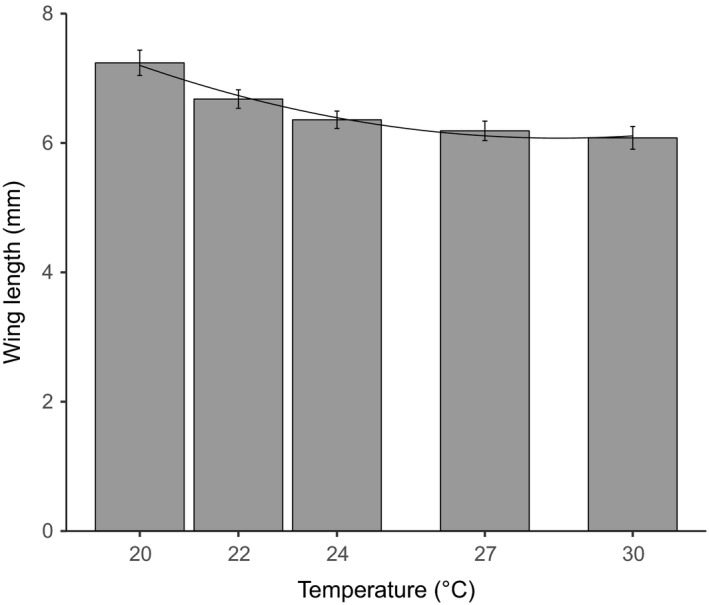
Temperature effects on the wing length (a proxy for adult body size). Error bars are ±1 *SEM*. Fitted lines show the predicted values from the fitted quadratic model

Increasing temperature significantly shortens the development time from egg to pupation and from pupation to eclosion, with a quadratic model giving the best fit to the data in both cases (Figure 1, likelihood ratio tests on fitted models, egg‐to‐pupa time, temperature LR = 8.00, 1*df*,* p* = .005, temperature^2^ LR = 6.95, 1*df*,* p* = .008; pupation to eclosion time temperature LR = 6.95, 1*df*,* p* = .008, temperature^2^ LR = 4.31, 1*df*,* p* = .038, Sections 2.1 & 2.2 in Appendix S1). The significant quadratic terms arise because in both cases, the effect is felt more strongly at cooler temperatures, resulting in a curved relationship between temperature and development. Increasing temperature also had the effect of reducing body size, with the relationship between temperature and body size being steeper at cooler temperatures, once again requiring that a quadratic term be retained in the final model (Figure 2, likelihood ratio test, temperature LR = 11.87, 1*df*,* p* = .0006, temperature^2^ LR = 10.70, 1*df p* = .0010, Section 3 in Appendix S1).

**Figure 3:**
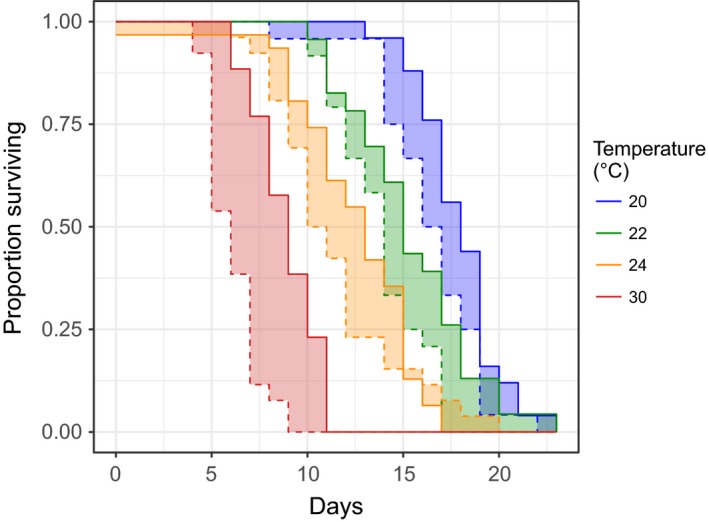
Survival of mated and unmated female moths at different temperatures. Solid lines indicate unmated moths and dashed lines mated ones. Color fills show the differences between mated and unmated at different temperatures. NB 27° treatment not shown for clarity, but it is intermediate between 24 and 30°C

Turning to the data for adult survival, both the act of mating and increasing temperature significantly reduced adult longevity in female *Plodia*. Mating costs became more pronounced at higher temperatures: Longevity declined by an average of 1.6 days, or 10% at 20 and 22°C, whereas at 30 °C mating reduced life expectancy by an average of 2.8 days, or 32%. This resulted in a significant interaction between the two factors (Figure 3, χ^2^ = 10.79, *p* = .001, 1*df*, Section 4 in Appendix S1).

**Figure 4:**
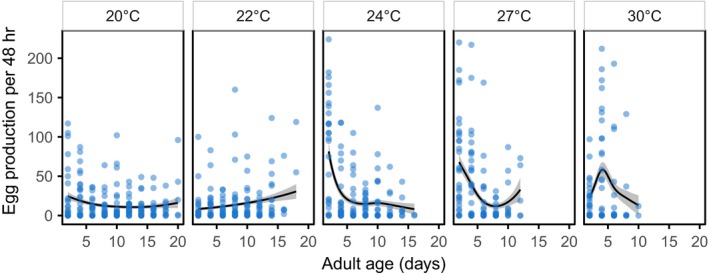
Number of eggs laid per 48 hr plus the fitted smoothers from the GAMM for each temperature. At 20°, the moths live for a long time and produce a few eggs every day. The moths at 22° have a similarly low oviposition rate but toward the end of their lives some of them increase the number of eggs laid (NB some of the 20° moths did this as well but some did not). Once the temperature reaches 24° and up oviposition is mostly early on in the animals’ lives, with large numbers of eggs laid in the first few days

The complex and divergent shapes relating oviposition rate to time at different temperatures made it necessary to analyze these data using a GAMM, which indicated significant temperature‐based differences in egg‐laying strategy (Figure 4). Marginal tests for the significance of individual smoothers all gave *p*‐values <.05 (Section 5.2 in Appendix S1), indicating significant differences between the shape of the relationship between temperature and oviposition for each treatment group. Moths reared in the two coldest temperatures had lower rates of egg production, but either maintained this rate of production or even increased it as they aged. The associated increase in longevity meant that these moths tended to produce a consistent number of eggs for a long period of time. Moths in warmer environments tended to have very high rates of egg production early in their lives, but as they aged, their egg production per unit time fell to values similar to those seen in the animals maintained in cooler environments. Interestingly, although there is a suggestion that lifetime reproductive success varies with temperature such that animals from intermediate temperatures have the highest reproductive output, this was not statistically significant (likelihood ratio test, temperature LR = 2.54, 1*df*,* p* = .11, temperature^2^ LR = 2.46, *p* = .12, 1*df*, Section 5.1 in Appendix S1), indicating that different strategies produce similar outcomes in terms of overall fecundity.

### 3.2 Immune assays

**Figure 5:**
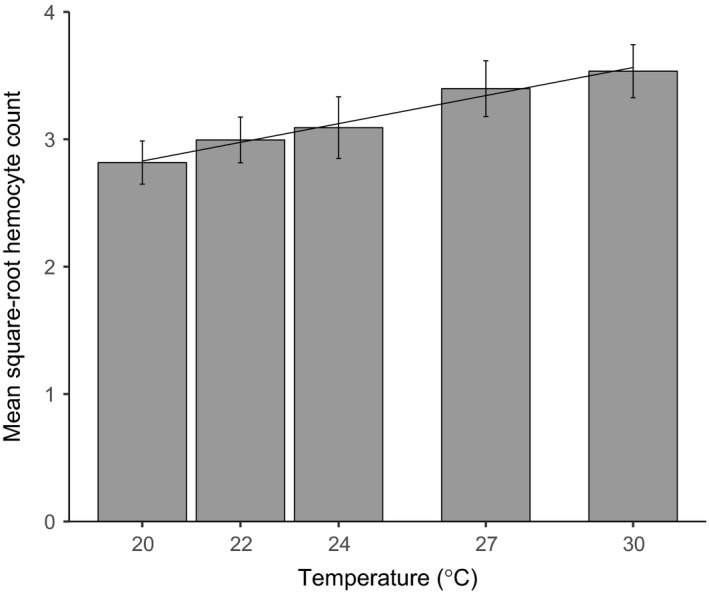
Temperature effects on hemocyte count. The bars show means and 95% confidence intervals for square‐root hemocyte count for each temperature treatment. The fitted line shows the predicted values from the model

Total hemocyte count increased significantly and linearly with increasing temperature (Figure 5, likelihood ratio test, temperature effect, LR = 14.151, *p* = .0002, 1*df*; Section 6.1 in Appendix S1). There was no significant effect of experimental block (χ^2^ = 0.055, *p* = .814, 1*df*) or larval weight (χ^2^ = 0.453, *p* = .501, 1*df*) on hemocyte count.

**Figure 6:**
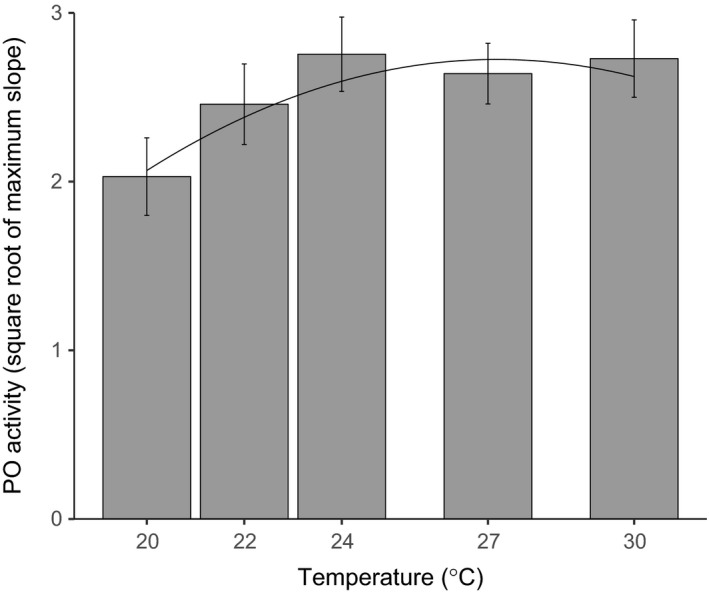
Phenoloxidase activity plotted against temperature. The line indicates predicted values from the fitted model. Error bars are 95% confidence intervals

PO activity also increased linearly with increasing temperature up to 24°C but leveled out across the warmer temperature treatments, leading to a final model with a quadratic term included (Figure 6, likelihood ratio test, temperature LR = 6.49, 1*df*,* p* = .011, temperature^2^ LR = 5.884, 1*df*,* p* = .015, Section 6.2 in Appendix S1). There was also a significant effect of larval weight (likelihood ratio test, LR = 12.435, 1*df*,* p* = .0004), with heavier larvae exhibiting lower levels of PO activity. There was no significant effect of experimental block (χ^2^ = 0.681, *p* = .409, 1*df*).

**Figure 7:**
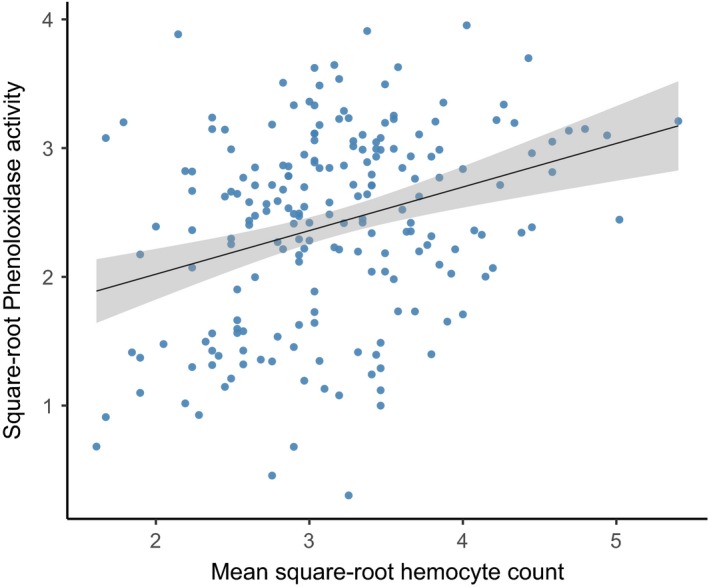
Square‐root PO activity plotted against square‐root hemocyte count. The line is for illustrative purposes only and shows the results of a simple linear regression through the data

Regression analysis showed a significant positive correlation between hemocyte counts and PO activity (Figure 7, *F*_1,198_ = 17.01, *p* < .0001), meaning that, while not explaining all the variation seen, larvae with more hemocytes also tended to have higher levels of PO activity.

### 3.3 Bacterial clearance

**Figure 8:**
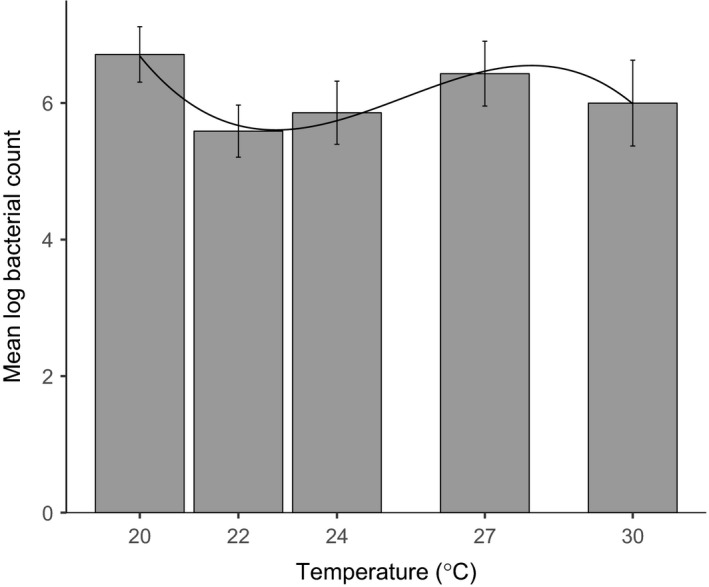
Temperature effects on bacterial clearance. The bars show means and 95% confidence intervals for log bacterial counts for each temperature treatment. The fitted line shows the predicted values from the fitted cubic model

The relationship between bacterial clearance and temperature was complex and best described by a model that included a cubic term (Figure 8, likelihood ratio test, temperature LR = 9.44, *p* = .002, temperature^2^ LR = 9.31, *p* = .002, temperature^3^ LR = 9.18, *p* = .002, all on 1*df*, Section 6.3 in Appendix S1). Larvae reared at 22°C and 24°C were better able to clear a bacterial infection than those reared at either the coldest (20°C) or hottest (27°C and 30°C) temperatures. There was no significant effect of block or larval weight.

### 3.4 Virus resistance

**Figure 9:**
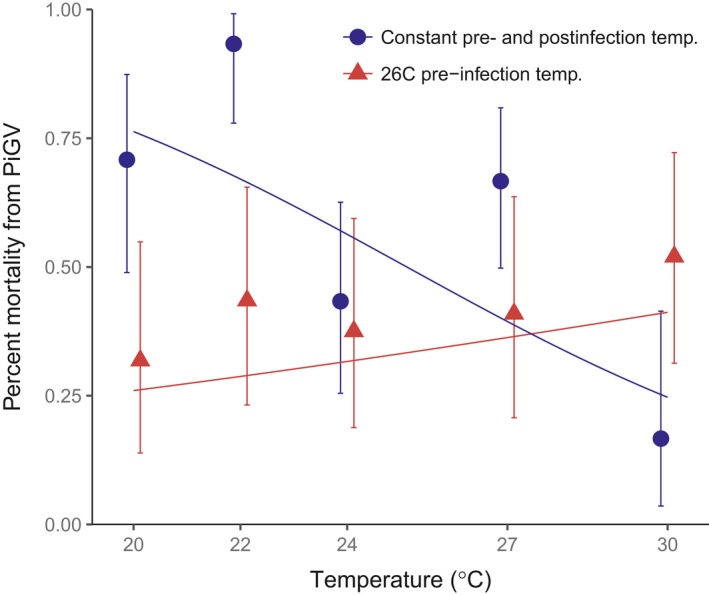
Ability of *Plodia* to resist infection with PiGV under two different temperature regimes. Moths experienced either a constant pre‐ and postinfection temperature or were raised at 26°C and only separated into temperature treatment groups after being dosed with PiGV. Virus resistance declines with temperature, but only when temperatures before and after infection are both manipulated. When only the temperatures after infection are manipulated, there is little effect of temperature. Lines indicate predicted values from the fitted model. Error bars are ±1 binomial *SEM*

There was a significant interaction between the pre‐ and postinfection temperatures (Figure 9, likelihood ratio test, LR = 14.00, *p* = .0002, 1*df*, Section 6.4 in Appendix S1), indicating that the relationship between temperature and virus resistance depends on the temperature experienced prior to infection, with larvae reared at cooler temperatures less able to resist infection. Of particular interest, larvae continuously reared at 30°C showed a 35% decrease in PiGV infection compared to larvae that were reared at 30°C only after infection.

## 4 DISCUSSION

All life‐history and immune measures and disease resistance assays were strongly influenced by environmental temperature. Rates of growth and development responded in a manner consistent with both previous studies (Johnson, Wofford, & Whitehand, 1992) and the temperature‐size rule (TSR, commonly used to describe the growth response of insects: Atkinson, 1994; but see also Angilletta & Dunham, 2003; Aguilar‐Alberola & Mesquita‐Joanes, 2014), with both measures accelerating linearly with increasing environmental temperature, resulting in individuals maturing faster but with a smaller body size (Atkinson & Sibly, 1997) and reduced survival time.

As temperature increased, mated females changed their egg‐laying strategy dramatically. Whereas low‐temperature females produced low numbers of eggs early in their lives, either maintaining or even increasing oviposition rate as they became older, females kept at 24°C and above produced most of their eggs in the first few days of adult life. The cost of this rapid oviposition appears to be a particularly strong effect of mating on lifespan, as shown by the significant interaction between mating status and temperature (Figure 1c); high‐temperature females follow a “live fast, die young” strategy whereas low‐temperature females reproduce at a more consistent rate and experience less of a reduction in lifespan.

Although females at warmer temperatures seem to be investing heavily in short‐term reproduction, there is no indication that they are trading off resources for this against immunity. On the contrary, both of our measures of the immune response increased with temperature, although in different ways. Hemocyte counts increased linearly, but PO activity exhibited a curved relationship such that there was little increase in activity above 24°C. These two components of the immune system therefore have different thermal sensitivity profiles (Thomas & Blanford, 2003), and consequently, the thermal sensitivity of parasite resistance in *Plodia* is likely to vary depending on which specific components of the immune response are most important in determining resistance to a specific pathogen. This is consistent with our results from bacterial and viral challenge: Whereas the mortality from viral infection declined with increasing rearing temperature, bacterial clearance showed a complex relationship with temperature, with the fewest bacteria remaining in animals reared at 22 and 24°C, but relatively low numbers of bacteria at 30°C as well.

Phenoloxidase plays a role in many physiological functions, including those involved in development (Bai, Xie, Shen, Wei, & Wang, 2014). As such, despite controlling for larval instar and body mass, the variation in developmental rates between the temperature treatments may be associated with intrinsic changes in active PO levels that have nothing to do with immune function (Lu et al., 2014). Interestingly, we found that heavier larvae had lower levels of PO activity regardless of rearing temperature, suggesting an inherent trade‐off between enzyme activity and growth and development. However, these data were recorded in unchallenged larvae. A positive correlation between PO activity and body size has been shown in vine moth larvae following a wounding challenge (Vogelweith, Thiéry, Moret, & Moreau, 2013), suggesting that larger individuals may have more resources to invest in immunity when a response is required.

Based on previous studies (Bauerfeind & Fischer, 2014; Murdock et al., 2012; Triggs & Knell, 2012), hemocyte density was expected to decrease at the higher temperature treatments. Instead, we saw numbers increase linearly with increasing temperature, something that has previously only been shown in *Plodia* when larvae are under additional environmental stressors (Triggs & Knell, 2012). This increase is unlikely to be due to dehydration effects, as we recorded no noticeable decrease in the total hemolymph volume available for collection from individuals raised at warmer temperatures. Higher temperatures might increase the rate of hemocyte production or multiplication or cause hemocytes to detach from tissue surfaces, so increasing the number in circulation (Pandey, Mishra, Kumar, Singh, & Prasad, 2010). Higher temperatures might also alter the relative abundance of cell types within the total hemocyte count (Pandey et al., 2010), with potential consequences for immune capacity.

Working on the assumption that higher levels of PO activity and hemocyte numbers correspond to increased immune capacity (Contreras‐Garduño et al., 2007; Pauwels et al., 2010; Poyet et al., 2013; Prevost & Eslin, 1998; Reeson et al., 1998; Valadez‐Lira et al., 2012) in *Plodia*, and that the potential immune response correlates with the realized immune response (Ferguson et al., 2016), we expected to see a decrease in bacterial populations with increasing temperature. We found no direct correlation between temperature and bacterial resistance, however, with larvae raised at 22–24°C being the most effective at clearing bacterial infections, despite these temperatures corresponding to the medium potential immune capacity in unchallenged larvae. One possible explanation for this is that bacterial clearance was assessed 24 hr after challenge, by which time the induced immune system should also be activated (Haine, Moret, Siva‐Jothy, & Rolff, 2008) and production of antimicrobial peptides upregulated. These components of the induced immune system could trade‐off with constitutive measures (Cotter, Kruuk, & Wilson, 2004; Cotter et al., 2008) or have very different thermal sensitivities to the innate components that we measured (Muturi, 2013; Muturi, Nyakeriga, & Blackshear, 2012), resulting in a reduction in the effectiveness of larval resistance. The impact of temperature on pathogen population dynamics is also likely to be important in determining the thermal sensitivity of infection. *E. coli* is usually cultured at 37°C in the laboratory, and growth rate should increase linearly with temperature within the range tested here (Ratkowsky et al., 1982). Faster rates of bacterial growth at the higher temperatures may therefore overwhelm the host immune response, resulting in a degree of unchecked bacterial proliferation.

Resistance to orally transmitted granulosis virus infection begins with a suite of protective mechanisms in the gut (Saejeng, Siva‐Jothy, & Boots, 2011). Once PiGV has breached gut defenses, however, hemocytes play an important role in removing virus particles from the hemocoel (Begon et al., 1993), and consequently, hemocyte abundance may positively correlate with PiGV resistance. Conversely, there is no evidence that PO activity plays a role in resistance to PiGV infections in *Plodia* (Saejeng, Tidbury, Siva‐Jothy, & Boots, 2010). Temperature effects on hemocyte densities in third‐instar larvae were not measured, but a linear increase with temperature similar to that seen in fifth‐instar larvae would equip hosts with a greater potential to phagocytose virus particles at higher temperatures (Begon et al., 1993), consistent with the increase in virus resistance in larvae kept at higher temperatures prior to infection.

In contrast to the larvae that were maintained at a constant temperature throughout the experiment, we saw no effect of postinfection temperature on virus resistance in those larvae kept at 26°C prior to infection. This could indicate that the changes in immune response leading to higher virus resistance only develop when an animal has experienced a particular temperature for several days. Alternatively, the increased rate of larval development associated with higher pre‐infection temperatures may lead to physiological changes such as faster rates of food passage through the gut (Yang & Joern, 1994) or increased rates of gut cell sloughing (Cory & Myers, 2003), both of which could prevent the establishment of a viral infection. In this case, postinfection temperature might have very little impact on viral population dynamics as the trajectory of the infection has already been established.

Overall, our results show that the effects of temperature change on life history and on disease resistance are complex and, in the case of disease resistance, pathogen‐specific (Adamo & Lovett, 2011). Difficulties in predicting outcomes lie not only in the need to understand the direct impact of temperature on individual components, but to also review the system as a whole, taking into account the dynamic nature of the selective pressures acting on life‐history trade‐offs across time (Alto & Bettinardi, 2013; Blanford, Thomas, Pugh, & Pell, 2003; Holland & Bourke, 2015; Laughton, Boots, & Siva‐Jothy, 2011). For example, higher temperatures may increase voltinism, potentially enabling host populations to rapidly adapt to thermal changes and infecting pathogens (Overgaard & Sørensen, 2008), but this may be tempered by reduced survival under temperature stress.

As a key component of terrestrial ecosystems, the sensitivity of insects to temperature likely has massive consequences for future ecosystem functioning. The potential alterations to the ecology of global pests such as *Plodia* by climate change effects are of major ecological and commercial importance. Likewise, the potential alterations to insect host–pathogen relationships have important implications for public health, biopesticide development, and conservation. Given the capacity for temperature to drive host–pathogen population dynamics and disease resistance, in‐depth consideration of these complex interactions is necessary to successfully predict ecological outcomes.

## CONFLICT OF INTEREST

None declared.

## DATA ACCESSIBILITY

Data files have been included with this submission to be published as supporting information.

## AUTHOR CONTRIBUTIONS

AL and RK conceived the ideas and designed the methodology; AL and CO collected the data; AL and RK analyzed the data; AL and RK led the writing of the manuscript. All authors contributed critically to the drafts and gave final approval for publication.

## References

[ece33506-bib-0001] Adamo, S. A. , & Lovett, M. M. E. (2011). Some like it hot: The effects of climate change on reproduction, immune function and disease resistance in the cricket *Gryllus* texensis. The Journal of Experimental Biology, 214, 1997–2004. 21613515 10.1242/jeb.056531

[ece33506-bib-0002] Aguilar‐Alberola, J. A. , & Mesquita‐Joanes, F. (2014). Breaking the temperature‐size rule: Thermal effects on growth, development and fecundity of a crustacean from temporary waters. Journal of Thermal Biology, 42, 15–24. 24802144 10.1016/j.jtherbio.2014.02.016

[ece33506-bib-0003] Alto, B. W. , & Bettinardi, D. (2013). Temperature and dengue virus infection in mosquitoes: Independent effects on the immature and adult stages. The American Journal of Tropical Medicine and Hygiene, 88, 497–505. 23382163 10.4269/ajtmh.12-0421 PMC3592531

[ece33506-bib-0004] Angilletta, M. J. , & Dunham, A. E. (2003). The temperature‐size rule in ectotherms: Simple evolutionary explanations may not be general. American Naturalist, 162, 332–342. 10.1086/377187 12970841

[ece33506-bib-0005] Angilletta, M. J. , Huey, R. B. , & Frazier, M. R. (2010). Thermodynamic effects on organismal performance: Is hotter better? Physiological and Biochemical Zoology, 83, 197–206. 20001251 10.1086/648567

[ece33506-bib-0006] Arbogast, R. T. (2007). A wild strain of *Plodia interpunctella* (Hübner) (Lepidoptera: Pyralidae) from farm‐stored maize in South Carolina: Effect of temperature on mating, survival, and fecundity. Journal of Stored Products Research, 43, 503–507.

[ece33506-bib-0007] Atkinson, D. (1994). Temperature and organism size: A biological law for ectotherms? Advances in Ecological Research, 25, 1–58.

[ece33506-bib-0008] Atkinson, D. , & Sibly, R. M. (1997). Why are organisms usually bigger in colder environments? Making sense of a life history puzzle. Trends in Ecology & Evolution, 12, 235–239. 21238056 10.1016/s0169-5347(97)01058-6

[ece33506-bib-0009] Bai, P.‐P. , Xie, Y.‐F. , Shen, G.‐M. , Wei, D.‐D. , & Wang, J.‐J. (2014). Phenoloxidase and its zymogen are required for the larval‐pupal transition in *Bactrocera dorsalis* (Diptera: Tephritidae). Journal of Insect Physiology, 71, 137–146. 25450426 10.1016/j.jinsphys.2014.10.013

[ece33506-bib-0010] Barnes, A. I. , & Siva‐Jothy, M. T. (2000). Density‐dependent prophylaxis in the mealworm beetle *Tenebrio molitor* L. (Coleoptera: Tenebrionidae): Cuticular melanization is an indicator of investment in immunity. Proceeding of the Royal Society B: Biological Sciences, 267, 177–182. 10.1098/rspb.2000.0984 PMC1690519 10687824

[ece33506-bib-0011] Bauerfeind, S. S. , & Fischer, K. (2014). Integrating temperature and nutrition ‐ environmental impacts on an insect immune system. Journal of Insect Physiology, 64, 14–20. 24636910 10.1016/j.jinsphys.2014.03.003

[ece33506-bib-0012] Beeman, S. C. , Wilson, M. E. , Bulla, L. A. , & Consigli, R. A. (1983). Structural characterization of the hemocytes of *Plodia interpunctella* . Journal of Morphology, 175, 1–16. 10.1002/jmor.1051750102 30064183

[ece33506-bib-0013] Begon, M. , Daud, K. , Young, P. , & Howells, R. E. (1993). The invasion and replication of a granulosis virus in the Indian meal moth, *Plodia interpunctella*: An electron microscope study. Journal of Invertebrate Pathology, 61, 281–295.

[ece33506-bib-0014] Blanford, S. , Thomas, M. B. , Pugh, C. , & Pell, J. K. (2003). Temperature checks the Red Queen? Resistance and virulence in a fluctuating environment. Ecology Letters, 6, 2–5.

[ece33506-bib-0015] Boots, M. , & Begon, M. (1994). Resource limitation and the lethal and sublethal effects of a viral pathogen in the Indian meal moth, *Plodia interpunctella* . Ecological Entomology, 19, 319–326.

[ece33506-bib-0016] Boots, M. , & Roberts, K. E. (2012). Maternal effects in disease resistance: Poor maternal environment increases offspring resistance to an insect virus. Proceedings of the Royal Society B: Biological Sciences, 279, 4009–4014. 22833270 10.1098/rspb.2012.1073 PMC3427573

[ece33506-bib-0017] Busso, J. P. , Blanckenhorn, W. U. , & González‐Tokman, D. (2017). Healthier or bigger? Trade‐off mediating male dimorphism in the black scavenger fly *Sepsis thoracica* (Diptera: Sepsidae). Ecological Entomology, 42, 517–525.

[ece33506-bib-0018] Castillo, J. , Brown, M. R. , & Strand, M. R. (2011). Blood feeding and insulin‐like peptide 3 stimulate proliferation of hemocytes in the mosquito *Aedes aegypti* . PLoS Pathogens, 7, e1002274. 21998579 10.1371/journal.ppat.1002274 PMC3188524

[ece33506-bib-0019] Catalán, T. , Niemeyer, H. , & Kalergis, A. (2012). Interplay between behavioural thermoregulation and immune response in mealworms. Journal of Insect Physiology, 58, 1450–1455. 22985859 10.1016/j.jinsphys.2012.08.011

[ece33506-bib-0020] Cerenius, L. , Lee, B. L. , & Söderhäll, K. (2008). The proPO‐system: Pros and cons for its role in invertebrate immunity. Trends in Immunology, 29, 263–271. 18457993 10.1016/j.it.2008.02.009

[ece33506-bib-0021] Cerenius, L. , & Söderhäll, K. (2004). The prophenoloxidase‐activating system in invertebrates. Immunological Reviews, 198, 116–126. 15199959 10.1111/j.0105-2896.2004.00116.x

[ece33506-bib-0022] Chown, S. L. , & Nicolson, S. W. (2004). Insect physiological ecology: Mechanisms and patterns. New York: Oxford University Press Inc..

[ece33506-bib-0023] Ciota, A. T. , Matacchiero, A. C. , Kilpatrick, A. M. , & Kramer, L. D. (2014). The effect of temperature on life history traits of *Culex* mosquitoes. Journal of Medical Entomology, 51, 55–62. 24605453 10.1603/me13003 PMC3955846

[ece33506-bib-0024] Clissold, F. J. , & Simpson, S. J. (2015). Temperature, food quality and life history traits of herbivorous insects. Current Opinion in Insect Science, 11, 63–70. 28285760 10.1016/j.cois.2015.10.011

[ece33506-bib-0025] Contreras‐Garduño, J. , Lanz‐Mendoza, H. , & Córdoba‐Aguilar, A. (2007). The expression of a sexually selected trait correlates with different immune defense components and survival in males of the American rubyspot. Journal of Insect Physiology, 53, 612–621. 17451742 10.1016/j.jinsphys.2007.03.003

[ece33506-bib-0026] Cory, J. S. , & Myers, J. H. (2003). The ecology and evolution of insect baculoviruses. Annual Review of Ecology, Evolution and Systematics, 34, 239–272.

[ece33506-bib-0027] Cotter, S. C. , Kruuk, L. , & Wilson, K. (2004). Costs of resistance: Genetic correlations and potential trade‐offs in an insect immune system. Journal of Evolutionary Biology, 17, 421–429. 15009275 10.1046/j.1420-9101.2003.00655.x

[ece33506-bib-0028] Cotter, S. C. , Myatt, J. P. , Benskin, C. M. H. , & Wilson, K. (2008). Selection for cuticular melanism reveals immune function and life‐history trade‐offs in *Spodoptera littoralis* . Journal of Evolutionary Biology, 21, 1744–1754. 18691239 10.1111/j.1420-9101.2008.01587.x

[ece33506-bib-0029] Elliot, S. L. , Blanford, S. , & Thomas, M. B. (2002). Host‐pathogen interactions in a varying environment: Temperature, behavioural fever and fitness. Proceedings of the Royal Society B: Biological Sciences, 269, 1599–1607. 12184830 10.1098/rspb.2002.2067 PMC1691072

[ece33506-bib-0030] Elrod‐Erickson, M. , Mishra, S. , & Schneider, D. (2000). Interactions between the cellular and humoral immune responses in *Drosophila* . Current Biology: CB, 10, 781–784. 10898983 10.1016/s0960-9822(00)00569-8

[ece33506-bib-0031] Eslin, P. , & Prevost, G. (1996). Variation in *Drosophila* concentration of haemocytes associated with different ability to encapsulate *Asobara tabinda* larval parasitoid. Journal of Insect Physiology, 42, 549–555.

[ece33506-bib-0032] Ferguson, L. V. , Heinrichs, D. E. , & Sinclair, B. J. (2016). Paradoxical acclimation responses in the thermal performance of insect immunity. Oecologia, 181, 77–85. 26846428 10.1007/s00442-015-3529-6

[ece33506-bib-0033] Freitak, D. , Wheat, C. , Heckel, D. , & Vogel, H. (2007). Immune system responses and fitness costs associated with consumption of bacteria in larvae of *Trichoplusia ni* . BMC Biology, 5, 56. 18154650 10.1186/1741-7007-5-56 PMC2235825

[ece33506-bib-0034] Fuller, C. A. , Postava‐Davignon, M. A. , West, A. , & Rosengaus, R. B. (2011). Environmental conditions and their impact on immunocompetence and pathogen susceptibility of the Caribbean termite *Nasutitermes acajutlae* . Ecological Entomology, 36, 459–470.

[ece33506-bib-0035] Giles, K. L. , Hellmich, R. L. , Iverson, C. T. , & Lewis, L. C. (2000). Effects of transgenic *Bacillus thuringiensis* maize grain on B. thuringiensis‐susceptible *Plodia interpunctella* (Lepidoptera: Pyralidae). Journal of Economic Entomology, 93, 1011–1016. 10902364 10.1603/0022-0493-93.3.1011

[ece33506-bib-0036] Gillespie, J. P. , Kanost, M. R. , & Trenczek, T. (1997). Biological mediators of insect immunity. Annual Review Entomology, 42, 611–643. 10.1146/annurev.ento.42.1.611 9017902

[ece33506-bib-0037] González‐Santoyo, I. , & Córdoba‐Aguilar, A. (2012). Phenoloxidase: A key component of the insect immune system. Entomologia Experimentalis et Applicata, 142, 1–16.

[ece33506-bib-0038] Haine, E. R. , Moret, Y. , Siva‐Jothy, M. T. , & Rolff, J. (2008). Antimicrobial defense and persistent infection in insects. Science, 322, 1257–1259. 19023083 10.1126/science.1165265

[ece33506-bib-0039] Hillyer, J. F. (2016). Insect immunology and hematopoiesis. Developmental and Comparative Immunology, 58, 102–118. 26695127 10.1016/j.dci.2015.12.006 PMC4775421

[ece33506-bib-0040] Holland, J. G. , & Bourke, A. F. G. (2015). Colony and individual life‐history responses to temperature in a social insect pollinator. Functional Ecology, 29, 1209–1217.

[ece33506-bib-0041] Horowitz, N. H. , & Shen, S. C. (1952). *Neurospora* tyrosinase. Journal of Biological Chemistry, 197, 513–520. 12981082

[ece33506-bib-0042] Johnson, J. A. , Wofford, P. L. , & Whitehand, L. C. (1992). Effect of diet and temperature on development rates, survival, and reproduction of the Indianmeal moth (Lepidoptera: Pyralidae). Journal of Economic Entomology, 85, 561–566.

[ece33506-bib-0043] Karl, I. , Stoks, R. , De Block, M. , Janowitz, S. A. , & Fischer, K. (2011). Temperature extremes and butterfly fitness: Conflicting evidence from life history and immune function. Global Change Biology, 17, 676–687.

[ece33506-bib-0044] Körner, M. , Vogelweith, F. , Foitzik, S. , & Meunier, J. (2017). Condition‐dependent trade‐off between weapon size and immunity in males of the European Earwig. Scientific Reports, 7, 7988. 28801629 10.1038/s41598-017-08339-6 PMC5554132

[ece33506-bib-0045] Kraaijeveld, A. R. , & Godfray, H. (2001). Basis of the trade–off between parasitoid resistance and larval competitive ability in *Drosophila melanogaster* . Proceedings of the Royal Society of London Series B: Biological Sciences, 268, 259–261. 11217895 10.1098/rspb.2000.1354 PMC1088600

[ece33506-bib-0046] Lachenicht, M. W. , Clusella‐Trullas, S. , Boardman, L. , Le Roux, C. , & Terblanche, J. S. (2010). Effects of acclimation temperature on thermal tolerance, locomotion performance and respiratory metabolism in *Acheta domesticus* L. (Orthoptera: Gryllidae). Journal of Insect Physiology, 56, 822–830. 20197070 10.1016/j.jinsphys.2010.02.010

[ece33506-bib-0047] Laughton, A. M. , Boots, M. , & Siva‐Jothy, M. T. (2011). The ontogeny of immunity in the honey bee, *Apis mellifera* L. following an immune challenge. Journal of Insect Physiology, 57, 1023–1032. 21570403 10.1016/j.jinsphys.2011.04.020

[ece33506-bib-0048] Littlefair, J. E. , Laughton, A. M. , & Knell, R. J. (2017). Maternal pathogen exposure causes diet‐and pathogen‐specific transgenerational costs. Oikos, 126, 82–90.

[ece33506-bib-0049] Lu, A. , Zhang, Q. , Zhang, J. , Yang, B. , Wu, K. , Xie, W. , … Ling, E. (2014). Insect prophenoloxidase: The view beyond immunity. Frontiers in Physiology, 5, 252. 25071597 10.3389/fphys.2014.00252 PMC4092376

[ece33506-bib-0050] Mohandass, S. , Arthur, F. H. , Zhu, K. Y. , & Throne, J. E. (2007). Biology and management of *Plodia interpunctella* (Lepidoptera: Pyralidae) in stored products. Journal of Stored Products Research, 43, 302–311.

[ece33506-bib-0051] Murdock, C. C. , Blanford, S. , Luckhart, S. , & Thomas, M. B. (2014). Ambient temperature and dietary supplementation interact to shape mosquito vector competence for malaria. Journal of Insect Physiology, 67, 37–44. 24911425 10.1016/j.jinsphys.2014.05.020 PMC4107084

[ece33506-bib-0052] Murdock, C. C. , Paaijmans, K. P. , Bell, A. S. , King, J. G. , Hillyer, J. A. N. F. , Read, A. F. , & Thomas, M. B. (2012). Complex effects of temperature on mosquito immune function. Proceedings of the Royal Society B: Biological Sciences, 279, 3357–3366. 22593107 10.1098/rspb.2012.0638 PMC3385736

[ece33506-bib-0053] Muturi, E. J. (2013). Larval rearing temperature influences the effect of malathion on *Aedes aegypti* life history traits and immune responses. Chemosphere, 92, 1111–1116. 23419321 10.1016/j.chemosphere.2013.01.055

[ece33506-bib-0054] Muturi, E. J. , Nyakeriga, A. , & Blackshear, M. (2012). Temperature‐mediated differential expression of immune and stress‐related genes in *Aedes aegypti* larvae. Journal of the American Mosquito Control Association, 28, 79–83. 22894117 10.2987/11-6194R.1

[ece33506-bib-0055] Na, J. H. , & Ryoo, M. I. (2000). The influence of temperature on development of *Plodia interpunctella* (Lepidoptera: Pyralidae) on dried vegetable commodities. Journal of Stored Products Research, 36, 125–129.

[ece33506-bib-0056] Nguyen, M. T. (2006). The effect of temperature on the growth of the bacteria *Escherichia coli* DH5α. Saint Martin's University Biology Journal, 1, 87–94.

[ece33506-bib-0057] Oluwafemi, A. R. , Rao, Q. , Wang, X. Q. , & Zhang, H. Y. (2009). Effect of *Bacillus thuringiensis* on *Habrobracon hebetor* during combined biological control of *Plodia interpunctella* . Insect Science, 16, 409–416.

[ece33506-bib-0058] Overgaard, J. , & Sørensen, J. G. (2008). Rapid thermal adaptation during field temperature variations in *Drosophila melanogaster* . Cryobiology, 56, 159–162. 18295194 10.1016/j.cryobiol.2008.01.001

[ece33506-bib-0059] Pamminger, T. , Steier, T. , & Tragust, S. (2016). High temperature and temperature variation undermine future disease susceptibility in a population of the invasive garden ant *Lasius neglectus* . Naturwissenschaften, 103, 46. 27206570 10.1007/s00114-016-1373-0

[ece33506-bib-0060] Pandey, J. P. , Mishra, P. K. , Kumar, D. , Singh, B. , & Prasad, B. C. (2010). Effect of temperature on hemocytic immune responses of tropical tasar silkworm, *Antheraea mylitta* D. Research Journal of Immunology, 3, 169–177.

[ece33506-bib-0061] Pauwels, K. , De Meester, L. , Decaestecker, E. , & Stoks, R. (2010). Phenoloxidase but not lytic activity reflects resistance against *Pasteuria ramosa* in *Daphnia magna* . Biology Letters, 7, 156–159. 20810432 10.1098/rsbl.2010.0634 PMC3030900

[ece33506-bib-0062] Pounds, J. A. , Bustamante, M. R. , Coloma, L. A. , Consuegra, J. A. , Fogden, M. P. L. , Foster, P. N. , … Young, B. E. (2006). Widespread amphibian extinctions from epidemic disease driven by global warming. Nature, 439, 161–167. 16407945 10.1038/nature04246

[ece33506-bib-0063] Poyet, M. , Havard, S. , Prevost, G. , Chabrerie, O. , Doury, G. , Gibert, P. , & Eslin, P. (2013). Resistance of *Drosophila suzukii* to the larval parasitoids *Leptopilina heterotoma* and *Asobara japonica* is related to haemocyte load. Physiological Entomology, 38, 45–53.

[ece33506-bib-0064] Prevost, G. , & Eslin, P. (1998). Hemocyte load and immune resistance to Asobara tabida are correlated in species of the *Drosophila melanogaster* subgroup. Journal of Insect Physiology, 44, 807–816. 12769876 10.1016/s0022-1910(98)00013-4

[ece33506-bib-0065] R Core Team . (2015). R: A language and environment for statistical computing. Vienna, Austria: R Foundation for Statistical Computing ISBN 3‐900051‐07‐0, URL http://www.R-project.org/.

[ece33506-bib-0066] Ratkowsky, D. A. , Olley, J. , McMeekin, T. A. , & Ball, A. (1982). Relationship between temperature and growth rate of bacterial cultures. Journal of Bacteriology, 149, 1–5. 7054139 10.1128/jb.149.1.1-5.1982 PMC216584

[ece33506-bib-0067] Reeson, A. F. , Wilson, K. , Gunn, A. , Hails, R. S. , & Goulson, D. (1998). Baculovirus resistance in the noctuid *Spodoptera exempta* is phenotypically plastic and responds to population density. Proceedings of the Royal Society B: Biological Sciences, 265, 1787–1791.

[ece33506-bib-0068] Ribeiro, C. , & Brehélin, M. (2006). Insect haemocytes: What type of cell is that? Journal of Insect Physiology, 52, 417–429. 16527302 10.1016/j.jinsphys.2006.01.005

[ece33506-bib-0069] Richards, S. L. , Anderson, S. L. , Lord, C. C. , & Tabachnick, W. J. (2012). Effects of virus dose and extrinsic incubation temperature on vector competence of *Culex nigripalpus* (Diptera: Culicidae) for St. Louis encephalitis virus. Journal of Medical Entomology, 49, 1502–1506. 23270182 10.1603/me12054 PMC3546324

[ece33506-bib-0070] Rolff, J. , & Siva‐Jothy, M. (2003). Invertebrate ecological immunology. Science, 301, 472–475. 12881560 10.1126/science.1080623

[ece33506-bib-0071] Saejeng, A. , Siva‐Jothy, M. T. , & Boots, M. (2011). Low cost antiviral activity of *Plodia interpunctella* haemolymph in vivo demonstrated by dose dependent infection. Journal of Insect Physiology, 57, 246–250. 21070782 10.1016/j.jinsphys.2010.10.005

[ece33506-bib-0072] Saejeng, A. , Tidbury, H. , Siva‐Jothy, M. T. , & Boots, M. (2010). Examining the relationship between hemolymph phenoloxidase and resistance to a DNA virus, Plodia interpunctella granulosis virus (PiGV). Journal of Insect Physiology, 56, 1232–1236. 20380834 10.1016/j.jinsphys.2010.03.025

[ece33506-bib-0073] Sait, S. M. , Begon, M. , & Thompson, D. J. (1994). The influence of larval age on the response of *Plodia interpunctella* to a granulosis virus. Journal of Invertebrate Pathology, 64, 107–110.7963644

[ece33506-bib-0074] Schmid‐Hempel, P. (2005). Evolutionary ecology of insect immune defenses. Annual Review of Entomology, 50, 529–551. 10.1146/annurev.ento.50.071803.130420 15471530

[ece33506-bib-0075] Schwenke, R. A. , Lazzaro, B. P. , & Wolfner, M. F. (2015). Reproduction‐immunity trade‐offs in insects. Annual Review of Entomology, 61, 239–256. 10.1146/annurev-ento-010715-023924 PMC5231921 26667271

[ece33506-bib-0076] Sternberg, E. D. , & Thomas, M. B. (2014). Local adaptation to temperature and the implications for vector‐borne diseases. Trends in Parasitology, 30, 115–122. 24513566 10.1016/j.pt.2013.12.010

[ece33506-bib-0077] Sutherland, J. P. , Bayliss, A. J. , & Braxton, D. S. (1995). Predictive modelling of growth of *Escherichia coli* O157:H7: The effects of temperature, pH and sodium chloride. International Journal of Food Microbiology, 25, 29–49. 7599029 10.1016/0168-1605(94)00082-h

[ece33506-bib-0078] Suwanchaichinda, C. , & Paskewitz, S. M. (1998). Effects of larval nutrition, adult body size, and adult temperature on the ability of *Anopheles gambiae* (Diptera: Culicidae) to melanize sephadex beads. Journal of Medical Entomology, 35, 157–161. 9538577 10.1093/jmedent/35.2.157

[ece33506-bib-0079] Therneau, T. (2015) Mixed effects Cox models. https://cran.r-project.org/web/packages/coxme/vignettes/coxme.pdf

[ece33506-bib-0080] Thomas, M. B. , & Blanford, S. (2003). Thermal biology in insect‐parasite interactions. Trends in Ecology & Evolution, 18, 344–350.

[ece33506-bib-0081] Triggs, A. , & Knell, R. J. (2012). Interactions between environmental variables determine immunity in the Indian meal moth *Plodia interpunctella* . Journal of Animal Ecology, 81, 386–394. 21999965 10.1111/j.1365-2656.2011.01920.x

[ece33506-bib-0082] Tzanakakis, M. E. (1959). An ecological study of the Indian‐meal moth *Plodia interpunctella* (Hübner) with emphasis on diapause. Hilgardia, 29, 205–246.

[ece33506-bib-0083] Valadez‐Lira, J. A. , Alcocer‐Gonzalez, J. M. , Damas, G. , Nuñez‐Mejía, G. , Oppert, B. , Rodriguez‐Padilla, C. , & Tamez‐Guerra, P. (2012). Comparative evaluation of phenoloxidase activity in different larval stages of four lepidopteran pests after exposure to *Bacillus thuringiensis* . Journal of Insect Science, 12, 80. 23414117 10.1673/031.012.8001 PMC3593704

[ece33506-bib-0084] Vogelweith, F. , Thiéry, D. , Moret, Y. , & Moreau, J. (2013). Immunocompetence increases with larval body size in a phytophagous moth. Physiological Entomology, 38, 219–225.

[ece33506-bib-0085] Ward, J. R. , Kim, K. , & Harvell, C. D. (2007). Temperature affects coral disease resistance and pathogen growth. Marine Ecology Progress Series, 329, 115–121.

[ece33506-bib-0086] Wilson, K. , Knell, R. , Boots, M. , & Koch‐Osborne, J. (2003). Group living and investment in immune defence: An interspecific analysis. Journal of Animal Ecology, 72, 133–143.

[ece33506-bib-0087] Wolinska, J. , & King, K. C. (2009). Environment can alter selection in host‐parasite interactions. Trends in Parasitology, 25, 236–244. 19356982 10.1016/j.pt.2009.02.004

[ece33506-bib-0088] Wood, S. N. (2006). Generalized additive models: An introduction with R. London: Chapman and Hall/CRC.

[ece33506-bib-0089] Yang, Y. , & Joern, A. (1994). Influence of diet quality, developmental stage, and temperature on food residence time in the grasshopper *Melanoplus differentialis* . Physiological Zoology, 67, 598–616.

[ece33506-bib-0090] Zuur, A. F. , Ieno, E. N. , Walker, N. , Saveliev, A. A. , & Smith, G. M. (2009). Mixed effects models and extensions in ecology with R. New York: Springer.

