# Supplementary material for: Responses to a warming world: Integrating life history, immune investment, and pathogen resistance in a model insect species
Source: Ecol Evol. 2017 Oct 16;7(22):9699–710. doi: 10.1002/ece3.3506 (PMC5696387; doi:10.1002/ece3.3506)
Supplement: Supplementary file 1 [file ECE3-7-9699-s001.pdf]

Analysis of Temperature effects on life history and immune traits

Rob Knell

6th September 2017

Contents

1 Preamble 1

2 Developmental time 2
- 2.1 Egg to pupa period 2
- 2.2 Pupal duration 3
- 2.3 Egg to adult 4

3 Body size and temperature 7

4 Survival 9

5 Oviposition and fertility 13
- 5.1 Total eggs laid 13
- 5.2 Eggs laid over time 14

6 Immunity measures and disease resistance 17
- 6.1 Haemocyte counts 17
- 6.2 Phenoloxidase activity 20
- 6.3 Correlation between haemocyte count and PO activity 24
- 6.4 Bacterial clearance 25
- 6.5 Virus resistance 28

1 Preamble

This document contains analysis and commentary for the data produced from the experiments described in "Responses to a warming world: integrating life history, immune investment and pathogen resistance in a model insect species"", authors Alice Laughton, Cian O'Connor and Rob Knell.

All data were analysed using R v3.4.0[1]. Linear mixed effects models were fitted to the majority of response variables, with temperature as a continuous explanatory variable and incubator as a random factor. Block and weight were fitted as covariates when appropriate. When exploratory analysis and inspection of diagnostic plots indicated a curved relationship between temperature and a response variable, polynomial models were fitted, and in a few cases the response variable was transformed when diagnostic plots indicated deviation from model assumptions. Most models used normal errors but virus infection was analysed using binomial errors. The data on survival were modelled using a mixed-effects Cox proportional hazards model fitted using the coxme package[2] with no censoring (data conformed to model assumptions).

The effect of temperature on egg production was analysed using a generalized additive mixed effects model (GAMM), fitted using the mgcv package. Individual moth ID was nested within incubator as the random factors and separate smoothers were fitted for each level of temperature.

[1] R Core Team (2017). R: A language and environment for statistical computing. R Foundation for Statistical Computing, Vienna, Austria. URL https://www.R-project.org/.

[2] Terry M. Therneau (2015). coxme: Mixed Effects Cox Models. R package version 2.2-5. https://CRAN.R-project.org/package=coxme

Mixed-effects models have to be used cautiously when the number of levels of a random factor is low (see, for example, http://glmm.wikidot.com/faq), and in this study there were five incubators, which is a sufficiently small number to sometimes cause problems during model fitting. These problems usually manifest as failure to converge, unrealistically large coefficient estimates or estimates of zero for the variance component associated with a random factor, and during the model fitting process we were alert to these potential issues. The only model in our analysis that showed any of these issues was the model fitted to the haemocyte count data, which gave an estimated variance of zero for the random factor. This model also displayed some positive skew in the distribution of residuals, and a square-root transformation of the response variable corrected both of these problems.

```
# Set working directory and load packages needed for analysis and visualisation
setwd("~/Dropbox/Temp gradient paper/Data analysis/")
library(ggplot2)
library(tidyr)
library(mgcv)
library(lmerTest)
library(dplyr)
```

2 Developmental time

2.1 Egg to pupa period

2.1.1 Deletion test on fitted model

```
######## Life history: developmental period

emergence <- read.csv("Laughton_et_al_dev_period.csv")

Box <- as.numeric(as.factor(emergence$Temp))

emergence <- data.frame(emergence, Box)

rm(Box)

pupationmod1 <- lmer(Day.egg.to.pup ~ Temp + I(Temp^2) + (1 | Box), data = emergence)

drop1(pupationmod1, test = "Chisq")
```

```
## Single term deletions
##
## Model:
## Day.egg.to.pup ~ Temp + I(Temp^2) + (1 | Box)
##           Df    AIC    LRT  Pr(Chi)
## <none>       1029.0
## Temp       1 1035.0 7.9937 0.004694 **
## I(Temp^2)  1 1033.9 6.9463 0.008399 **
## ---
## Signif. codes:  0 '***' 0.001 '**' 0.01 '*' 0.05 '.' 0.1 ' ' 1
```

2.1.2 Summary of fitted model

```
summary(pupationmod1)
```

```
## Linear mixed model fit by REML t-tests use Satterthwaite approximations
##   to degrees of freedom [lmerMod]
## Formula: Day.egg.to.pup ~ Temp + I(Temp^2) + (1 | Box)
##    Data: emergence
##
## REML criterion at convergence: 1024.5
##
## Scaled residuals:
##     Min      1Q  Median      3Q     Max
## -1.5663 -0.4563 -0.3202  0.2980  4.6480
##
## Random effects:
##  Groups   Name        Variance Std.Dev.
##  Box      (Intercept) 2.312    1.521
##  Residual             2.589    1.609
## Number of obs: 265, groups:  Box, 5
##
## Fixed effects:
##              Estimate Std. Error        df t value Pr(>|t|)
## (Intercept) 153.83518   41.34919   2.00340   3.720   0.0651 .
## Temp         -9.42522    3.35373   2.00310  -2.810   0.1065
## I(Temp^2)     0.16410    0.06684   2.00310   2.455   0.1333
## ---
## Signif. codes:  0 '***' 0.001 '**' 0.01 '*' 0.05 '.' 0.1 ' ' 1
##
## Correlation of Fixed Effects:
##           (Intr) Temp
## Temp      -0.998
## I(Temp^2)  0.993 -0.998
```

Egg to pupal period declines with temperature with a curved relationship between the two - the slope is steeper towards the cooler temperatures.

2.2 Pupal duration

2.2.1 Deletion test on fitted model

```
pupal.period <- emergence$Egg.to.ecols - emergence$Day.egg.to.pup

pupalmod1 <- lmer(pupal.period ~ Temp + I(Temp^2) + (1 | Box), data = emergence)

drop1(pupalmod1, test = "Chisq")
```

```
## Single term deletions
##
## Model:
## pupal.period ~ Temp + I(Temp^2) + (1 | Box)
##           Df    AIC    LRT  Pr(Chi)
## <none>       1073.7
## Temp       1 1078.7 6.9511 0.008377 **
## I(Temp^2)  1 1076.1 4.3145 0.037790 *
## ---
## Signif. codes:  0 '***' 0.001 '**' 0.01 '*' 0.05 '.' 0.1 ' ' 1
```

2.2.2 Summary of fitted model

```
summary(pupalmod1)
```

```
## Linear mixed model fit by REML t-tests use Satterthwaite approximations
##   to degrees of freedom [lmerMod]
## Formula: pupal.period ~ Temp + I(Temp^2) + (1 | Box)
##    Data: emergence
##
## REML criterion at convergence: 1074.6
##
## Scaled residuals:
##     Min      1Q  Median      3Q     Max
## -5.2073 -0.6379 -0.1475  0.4131  6.6495
##
## Random effects:
##  Groups   Name        Variance Std.Dev.
##  Box      (Intercept) 0.347    0.589
##  Residual             3.182    1.784
## Number of obs: 265, groups:  Box, 5
##
## Fixed effects:
##             Estimate Std. Error       df t value Pr(>|t|)
## (Intercept) 68.50835   17.16567  1.96280   3.991   0.0592 .
## Temp        -3.41200    1.39201  1.96110  -2.451   0.1363
## I(Temp^2)    0.04564    0.02774  1.96080   1.645   0.2441
## ---
## Signif. codes:  0 '***' 0.001 '**' 0.01 '*' 0.05 '.' 0.1 ' ' 1
##
## Correlation of Fixed Effects:
##           (Intr) Temp
## Temp      -0.998
## I(Temp^2)  0.993 -0.998
```

Pupal duration shows a similar pattern to egg to pupal period with steep declines with temperature at cooler temperatures and a shallower slope when temperatures are warmer.

2.3 Egg to adult

2.3.1 Deletion test on fitted model

```
eclosemod1 <- lmer(Egg.to.ecols ~ Temp + I(Temp^2) + (1 | Box), data = emergence)

drop1(eclosemod1, test = "Chisq")
```

```
## Single term deletions
##
## Model:
## Egg.to.ecols ~ Temp + I(Temp^2) + (1 | Box)
##           Df    AIC    LRT Pr(Chi)
## <none>       1051.0
## Temp       1 1056.8 7.7472 0.00538 **
## I(Temp^2)  1 1055.3 6.2510 0.01241 *
```

```
## ---
## Signif. codes:  0 '***' 0.001 '**' 0.01 '*' 0.05 '.' 0.1 ' ' 1
```

2.3.2 Summary of fitted model

```
summary(eclosemod1)
```

```
## Linear mixed model fit by REML t-tests use Satterthwaite approximations
##   to degrees of freedom [lmerMod]
## Formula: Egg.to.ecols ~ Temp + I(Temp^2) + (1 | Box)
##    Data: emergence
##
## REML criterion at convergence: 1044.5
##
## Scaled residuals:
##     Min      1Q  Median      3Q     Max
## -1.8931 -0.5653 -0.0946  0.6337  5.6415
##
## Random effects:
##  Groups   Name        Variance Std.Dev.
##  Box      (Intercept) 4.599    2.144
##  Residual             2.783    1.668
## Number of obs: 265, groups:  Box, 5
##
## Fixed effects:
##              Estimate Std. Error        df t value Pr(>|t|)
## (Intercept) 222.16381   58.03904   2.04080   3.828    0.060 .
## Temp        -12.82188    4.70746   2.04060  -2.724    0.110
## I(Temp^2)     0.20943    0.09383   2.04060   2.232    0.153
## ---
## Signif. codes:  0 '***' 0.001 '**' 0.01 '*' 0.05 '.' 0.1 ' ' 1
##
## Correlation of Fixed Effects:
##           (Intr) Temp
## Temp      -0.998
## I(Temp^2)  0.993 -0.998
```

```
## Plot of emergence periods

# Build data frame
pupal.period <- emergence$Egg.to.ecols - emergence$Day.egg.to.pup
Temp2 <- c(emergence$Temp, emergence$Temp)
Box2 <- c(emergence$Box, emergence$Box)
Period <- rep(c("Egg.to.pupation", "Pupal.period"), each = 265)
Duration <- c(emergence$Day.egg.to.pup, pupal.period)
emergence_stacked <- data.frame(Temp2, Box2, Period, Duration)

rm(pupal.period, Temp2, Box2, Period, Duration)


#### means and CIs

duration.means <- with(emergence_stacked, tapply(Duration, list(Temp2, Period), function(x) mean(x,
    na.rm = TRUE)))
```

```
duration.se <- with(emergence_stacked, tapply(Duration, list(Temp2, Period), function(x) sd(x)/sqrt(length(x))))

duration.95 <- with(emergence_stacked, tapply(Duration, list(Temp2, Period), function(x) (sd(x)/sqrt(length(x)) *
    qt(0.975, length(x) - 1))))

Temperature <- rep(c(20, 22, 24, 27, 30), times = 2)

Period <- factor(rep(c("Egg to pupation", "Pupation to eclosion"), each = 5))

Period <- relevel(Period, ref = "Pupation to eclosion")

Total.duration <- with(emergence, tapply(Egg.to.ecols, Temp, function(x) mean(x,
    na.rm = TRUE)))

emergence_means <- data.frame(Temperature, Period, c(duration.means[, 1], duration.means[,
    2]), c(duration.se[, 1], duration.se[, 2]), c(duration.95[, 1], duration.95[,
    2]), c(Total.duration, Total.duration))

names(emergence_means) <- c("Temperature", "Period", "Mean.duration", "SE", "CI95",
    "Total.duration")

dummy_duration <- ifelse(emergence_means$Period == "Egg to pupa", emergence_means$Mean.duration,
    emergence_means$Total.duration)

emergence_means <- data.frame(emergence_means, dummy_duration)

p1 <- ggplot(data = emergence_means, aes(x = Temperature, y = Mean.duration, fill = Period))
p1 <- p1 + geom_bar(stat = "identity", colour = "black", size = 0.2) + theme_classic() +
    scale_x_continuous(breaks = c(20, 22, 24, 27, 30))
p1 <- p1 + scale_fill_manual(values = c("grey90", "grey60"))
p1 <- p1 + geom_errorbar(aes(ymin = ifelse(Period == "Egg to pupation", Mean.duration -
    CI95, Total.duration - CI95), ymax = ifelse(Period == "Egg to pupation", Mean.duration +
    CI95, Total.duration + CI95)), colour = "black", width = 0.1, size = 0.2)
p1 <- p1 + ylab("Duration (days)") + xlab(expression(Temperature ~ degree * C)) +
    scale_y_continuous(limits = c(0, 50), expand = c(0, 0))
p1 <- p1 + theme(axis.line.y = element_line(colour = "black", size = 0.33), axis.line.x = element_line(colour = "blac[illegible]
    size = 0.33)) + theme(legend.position = c(0.8, 0.9))

p1 <- p1 + stat_function(fun = function(Temperature) 222.16381 - 12.82188 * Temperature +
    0.20943 * Temperature^2, size = 0.2)

# ggsave('fig1.pdf', width = 12, height = 10, units = 'cm')

p1
```

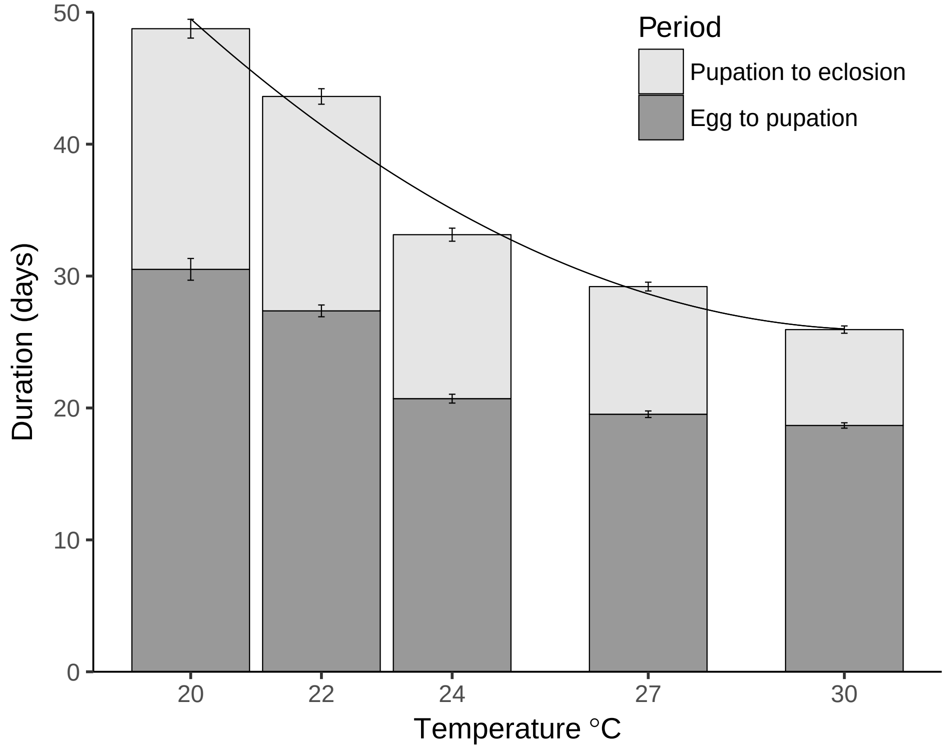


```
rm(list = ls())
```

Figure 1 Developmental times plotted against temperature. Error bars indicate 95% condfidence intervals. The line shows the predicted values from the fitted quadratic model for egg-to-adult development time.

3 Body size and temperature

3.0.1 Deletion test on fitted model

```
######### Life history: body size

wings <- read.csv("Laughton_et_al_wing_lengths.csv")

Box <- as.numeric(as.factor(wings$Temperature))

wings <- data.frame(wings, Box)

rm(Box)

wingmod <- lmer(Wing_length ~ Temperature + I(Temperature^2) + (1 | Box), data = wings)

drop1(wingmod, test = "Chisq")
```

```
## Single term deletions
##
## Model:
## Wing_length ~ Temperature + I(Temperature^2) + (1 | Box)
##                  Df    AIC    LRT   Pr(Chi)
```

```
## <none>                77.947
## Temperature       1 87.823 11.876 0.0005686 ***
## I(Temperature^2)  1 86.646 10.699 0.0010721 **
## ---
## Signif. codes:  0 '***' 0.001 '**' 0.01 '*' 0.05 '.' 0.1 ' ' 1
```

3.0.2 Summary of fitted model

```r
summary(wingmod)
```

```
## Linear mixed model fit by REML t-tests use Satterthwaite approximations
##   to degrees of freedom [lmerMod]
## Formula: Wing_length ~ Temperature + I(Temperature^2) + (1 | Box)
##    Data: wings
##
## REML criterion at convergence: 89.9
##
## Scaled residuals:
##      Min       1Q   Median       3Q      Max
## -2.51623 -0.59307  0.09134  0.64718  2.08305
##
## Random effects:
##  Groups   Name        Variance  Std.Dev.
##  Box      (Intercept) 0.0002778 0.01667
##  Residual             0.1189668 0.34492
## Number of obs: 100, groups:  Box, 5
##
## Fixed effects:
##                   Estimate Std. Error        df t value Pr(>|t|)
## (Intercept)      18.707224   2.123519  2.000000   8.810   0.0126 *
## Temperature      -0.886253   0.172238  2.000000  -5.146   0.0358 *
## I(Temperature^2)  0.015544   0.003433  2.000000   4.528   0.0455 *
## ---
## Signif. codes:  0 '***' 0.001 '**' 0.01 '*' 0.05 '.' 0.1 ' ' 1
##
## Correlation of Fixed Effects:
##             (Intr) Tmprtr
## Temperature -0.998
## I(Tmprtr^2)  0.993 -0.998
```

Body size, as indicated by wing length, shows a similar pattern to development time. Warmer temperatures lead to smaller adults, with the relationship between temperature and body size being steeper at cooler temperatures.

```r
wingmeans <- tapply(wings$Wing_length, wings$Temperature, function(x) mean(x, na.rm = TRUE))

wingCIs <- tapply(wings$Wing_length, wings$Temperature, function(x) (sd(x)/sqrt(length(x)) *
    qt(0.975, length(x) - 1)))

Temp <- c(20, 22, 24, 27, 30)

wing_means <- data.frame(Temp, wingmeans, wingCIs)

p1 <- ggplot(data = wing_means, aes(x = Temp, y = wingmeans))
p1 <- p1 + geom_bar(stat = "identity", fill = "grey60", colour = "black", size = 0.2) +
```

```
    theme_classic() + scale_x_continuous(breaks = c(20, 22, 24, 27, 30))
p1 <- p1 + geom_errorbar(aes(ymin = wingmeans - wingCIs, ymax = wingmeans + wingCIs),
    colour = "black", width = 0.1, size = 0.2)
p1 <- p1 + ylab("Wing length (mm)") + xlab(expression(Temperature ~ degree * C)) +
    scale_y_continuous(limits = c(0, 8), expand = c(0, 0))
p1 <- p1 + theme(axis.line.y = element_line(colour = "black", size = 0.33), axis.line.x = element_line(colour = "blac
    size = 0.33)) + theme(legend.position = c(0.8, 0.9))


p1 <- p1 + stat_function(fun = function(Temp) 18.707224 - 0.88625 * Temp + 0.015544 *
    (Temp^2), size = 0.2)
p1 <- p1 + xlab("Temperature (°C)") + ylab("Wing length (mm)")
p1
```

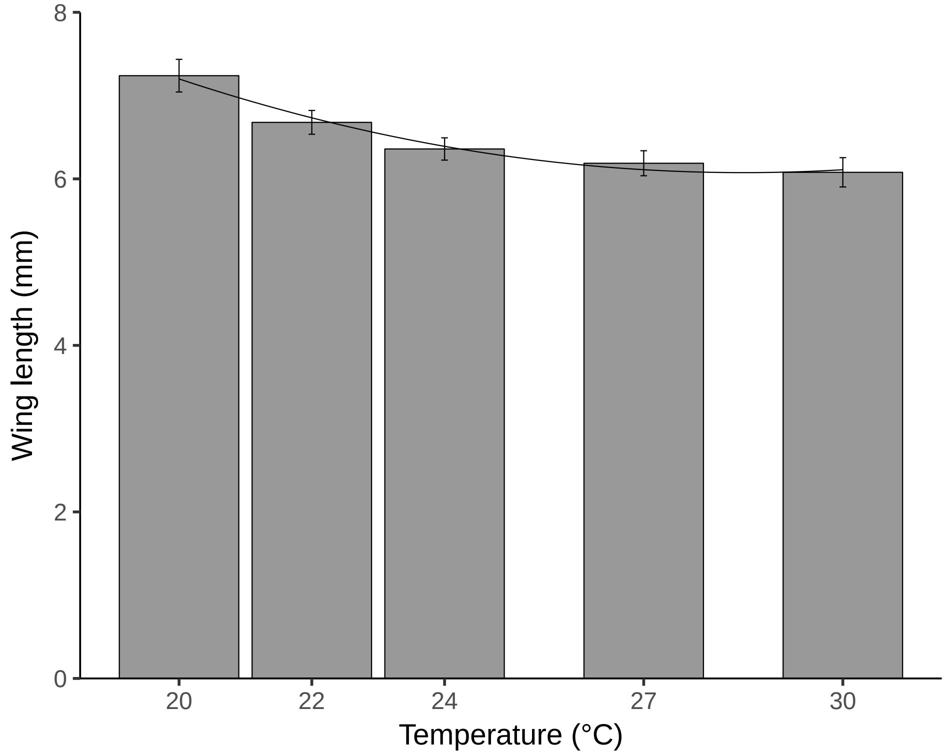


```
# ggsave('fig2.pdf', width = 12, height = 10, units = 'cm')

rm(list = ls())
```

Figure 2 Body size as indicated by wing length plotted against temperature. Error bars are 95% confidence intervals. The line shows the predicted valeus from the fitted quadratic model.

4 Survival

4.0.1 ANOVA table and summary of fitted model

```
############## Survival analysis
```

```r
library(survival)
library(coxme)
```

```
## Loading required package: bdsmatrix

##
## Attaching package: 'bdsmatrix'

## The following object is masked from 'package:base':
##
##     backsolve
```

```r
surv <- read.csv("Laughton_et_al_survival.csv")
Box <- as.numeric(as.factor(surv$Temp))
surv <- data.frame(surv, Box)
rm(Box)

survmod1 <- coxme(Surv(Death.time, Status) ~ Temp * Mated + (1 | Box), data = surv)

anova(survmod1)
```

```
## Analysis of Deviance Table
##  Cox model: response is Surv(Death.time, Status)
## Terms added sequentially (first to last)
##
##              loglik   Chisq Df Pr(>|Chi|)
## NULL        -1211.8
## Temp        -1103.8 215.832  1  < 2.2e-16 ***
## Mated       -1086.8  33.992  1  5.533e-09 ***
## Temp:Mated -1081.5  10.788  1   0.001022 **
## ---
## Signif. codes:  0 '***' 0.001 '**' 0.01 '*' 0.05 '.' 0.1 ' ' 1
```

```r
summary(survmod1)
```

```
## Cox mixed-effects model fit by maximum likelihood
##   Data: surv
##   events, n = 264, 264
##   Iterations= 5 32
##                     NULL Integrated    Fitted
## Log-likelihood -1211.758  -1081.452 -1081.438
##
##                   Chisq   df p    AIC    BIC
## Integrated loglik 260.61 4.00 0 252.61 238.31
##  Penalized loglik 260.64 3.01 0 254.61 243.84
##
## Model:  Surv(Death.time, Status) ~ Temp * Mated + (1 | Box)
## Fixed coefficients
##                  coef  exp(coef)   se(coef)     z      p
## Temp        0.3317337 1.39338180 0.03022047 10.98 0.0000
## Matedy     -2.3590153 0.09451324 0.96234270 -2.45 0.0140
## Temp:Matedy  0.1266846 1.13505891 0.03876694  3.27 0.0011
##
## Random effects
##  Group Variable  Std Dev      Variance
##  Box   Intercept 9.294735e-03 8.639211e-05
```

```r
#### Plot of survival curves

survmod2 <- survfit(Surv(Death.time, Status) ~ surv$Temp + surv$Mated, data = surv)  #Generate model

# Extract each factor combination in turn

start = 1  #Variable to indicate where in the dataset to start from for each level of strata


for (i in 1:length(survmod2$strata)) {

    end <- start + survmod2$strata[i] - 1  #Where to finish pulling data out for each value of strata

    Survival <- survmod2$surv[start:end]  #Proportions surviving at each time interval
    Times <- survmod2$time[start:end]  #Time intervals



    Survival <- c(1, Survival)  #It diesn't include the bit where nothing has died yet
    Times <- c(0, Times)  #or start time

    Time <- 0:23

    Survival2 <- rep(NA, times = 24)

    Survival2[match(Times, Time)] <- Survival

    temp1 <- data.frame(Time, Survival2)

    rm(Survival2)

    temp2 <- temp1 %>% fill(Survival2)

    Survival <- temp2$Survival2
    Times <- temp2$Time

    Survival <- rep(Survival, each = 2)  #Need two of each one to plot it properly
    Times <- rep(Times, each = 2)  #Two values for each time as well

    t1 <- length(Survival) - 1

    Survival <- Survival[-t1]  #remove the last number
    Times <- Times[-1]  #and the first one from the Times

    # plot(Times, Survival, type = 'l')

    # Temperature <- rep(substr(names(survmod2$strata)[i], start = 11, stop = 12),
    # Times = survmod2$strata[i]*2 +1) #Extract the temperature value from the name
    # for this stratum and make it into a variable
    Temperature <- rep(substr(names(survmod2$strata)[i], start = 11, stop = 12),
        47)  #Extract the temperature value from the name for this stratum and make it into a variable
    # Mated <- rep(substr(names(survmod2$strata)[i], start = 26, stop = 26), Times =
    # survmod2$strata[i]*2 +1) #Same again for whether the moths were mated
```

```
    Mated <- rep(substr(names(survmod2$strata)[i], start = 26, stop = 26), times = 47)  #Same again for whether the m

    # temp1 <- data.frame(Temperature, Mated, temp2)
    temp1 <- data.frame(Temperature, Mated, Times, Survival)

    if (i == 1)
        Surv_curves <- temp1

    if (i > 1)
        Surv_curves <- rbind(Surv_curves, temp1)  #Stick it all together in a data frame

    start <- start + survmod2$strata[i]

}

colnames(Surv_curves)[4] <- "Survival"



temp1 <- subset(Surv_curves, Mated == "y")
colnames(temp1)[4] <- "Survival_mated"
temp2 <- subset(Surv_curves, Mated == "n")
colnames(temp2)[4] <- "Survival_unmated"

Surv_curves2 <- data.frame(temp1, temp2$Survival_unmated)

colnames(Surv_curves2)[5] <- "Survival_unmated"

palette1 <- c("Blue", "Green4", "darkorange", "firebrick3")







p1 <- ggplot(data = subset(Surv_curves2, Temperature != "27"), aes(x = Times, y = Survival_unmated)) +
    xlab("Days") + ylab("Proportion surviving") + guides(color = guide_legend(title = "Temperature\n(°C)"))

p1 <- p1 + geom_ribbon(aes(x = Times, ymin = Survival_mated, ymax = Survival_unmated,
    fill = Temperature), alpha = 0.3, show.legend = FALSE) + theme_bw() + scale_fill_manual(values = palette1)

p1 <- p1 + geom_line(aes(x = Times, y = Survival_mated, colour = Temperature), linetype = "dashed",
    size = 0.4, show.legend = FALSE)

p1 <- p1 + geom_line(aes(x = Times, y = Survival_unmated, colour = Temperature),
    size = 0.4) + scale_color_manual(values = palette1)

p1
```

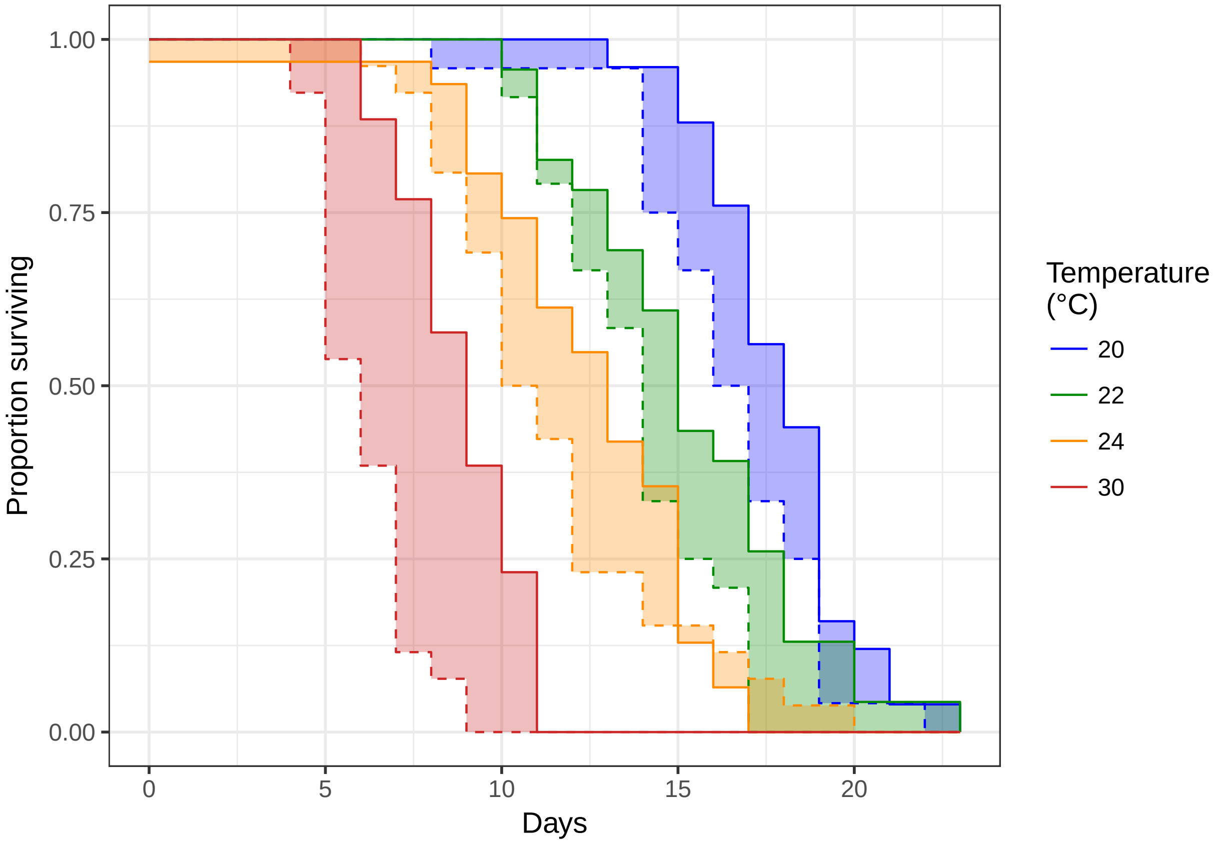


```
# ggsave('fig3.pdf', width = 14 , height = 10, units = 'cm')
```

Figure 3 Survival of mated and unmated moths at different temperatures. Solid lines indicate unmated moths and dashed lines mated ones. Colour fills show the differences between mated and unmated at different temperatures. NB 27° treatment not shown for clarity but it is intermediate between 24 and 30°C.

5 Oviposition and fertility

5.1 Total eggs laid

5.1.1 Deletion test on fitted model

```
eggs_time <- read.csv("Laught_et_al_egg_production.csv")

Box <- as.numeric(as.factor(eggs_time$Temp))

eggs_time <- data.frame(eggs_time, Box)

rm(Box)

egg.mod1 <- lmer(Total.egg ~ Temp + I(Temp^2) + (1 | Box), data = eggs_time, subset = Total.egg >
    0)

drop1(egg.mod1, test = "Chisq")
```

```
## Single term deletions
##
## Model:
## Total.egg ~ Temp + I(Temp^2) + (1 | Box)
##           Df    AIC    LRT Pr(Chi)
## <none>       1476.8
## Temp       1 1477.3 2.5375  0.1112
## I(Temp^2)  1 1477.3 2.4621  0.1166
```

5.1.2 Summary of fitted model

```r
summary(egg.mod1)
```

```
## Linear mixed model fit by REML t-tests use Satterthwaite approximations
##   to degrees of freedom [lmerMod]
## Formula: Total.egg ~ Temp + I(Temp^2) + (1 | Box)
##    Data: eggs_time
##  Subset: Total.egg > 0
##
## REML criterion at convergence: 1454.5
##
## Scaled residuals:
##      Min       1Q   Median       3Q      Max
## -2.10836 -0.64578 -0.04317  0.78127  2.19756
##
## Random effects:
##  Groups   Name        Variance Std.Dev.
##  Box      (Intercept)  681.7   26.11
##  Residual             5439.6   73.75
## Number of obs: 128, groups:  Box, 5
##
## Fixed effects:
##             Estimate Std. Error       df t value Pr(>|t|)
## (Intercept) -797.111    804.596    1.959  -0.991    0.428
## Temp          74.276     65.276    1.960   1.138    0.375
## I(Temp^2)     -1.453      1.302    1.964  -1.116    0.382
##
## Correlation of Fixed Effects:
##           (Intr) Temp
## Temp      -0.998
## I(Temp^2)  0.993 -0.998
```

Although there is a significant effect of Temperature on the total number of eggs laid when a simple linear model is used, including incubator as a random term means that this relationship ceases to be statistically significant.

5.2 Eggs laid over time

5.2.1 Summary of fitted GAMM

```r
######### Life history - egg laying strategy

# Convert data wide to long
```

```r
egglaying <- eggs_time[, c(1, 2, 12:21)]

egglaying <- gather(egglaying, Period, Egg.count, X1st48:X10th48)

egglaying$Period[which(egglaying$Period == "X10th48")] <- "Y10th48"

Days <- 2 * as.numeric(as.factor(egglaying$Period))

Box <- as.numeric(as.factor(egglaying$Temp))

egglaying <- data.frame(egglaying, Days, Box)

######## Fit GAMM

egg.gam <- gamm(Egg.count ~ as.factor(Temp) + s(Days, by = as.factor(Temp)), random = list(Sample = ~1,
    Box = ~1), data = egglaying, family = quasipoisson)
```

```
##
##  Maximum number of PQL iterations:  20
```

```r
# egg.gam2<-gamm(Egg.count~as.factor(Temp)+s(Days), random=~1|Box/Sample,
# data=egglaying, family=quasipoisson)


summary(egg.gam$gam)
```

```
##
## Family: quasipoisson
## Link function: log
##
## Formula:
## Egg.count ~ as.factor(Temp) + s(Days, by = as.factor(Temp))
##
## Parametric coefficients:
##                   Estimate Std. Error t value Pr(>|t|)
## (Intercept)         2.7240     0.1346  20.236  < 2e-16 ***
## as.factor(Temp)22  -0.1698     0.2006  -0.847  0.39748
## as.factor(Temp)24   0.3703     0.2007   1.845  0.06541 .
## as.factor(Temp)27   0.7643     0.2833   2.698  0.00714 **
## as.factor(Temp)30   0.2265     0.8436   0.269  0.78835
## ---
## Signif. codes:  0 '***' 0.001 '**' 0.01 '*' 0.05 '.' 0.1 ' ' 1
##
## Approximate significance of smooth terms:
##                             edf Ref.df      F  p-value
## s(Days):as.factor(Temp)20 2.010  2.010  4.598   0.0106 *
## s(Days):as.factor(Temp)22 1.000  1.000  7.340   0.0069 **
## s(Days):as.factor(Temp)24 2.939  2.939 23.328 5.17e-14 ***
## s(Days):as.factor(Temp)27 2.826  2.826 16.534 5.79e-07 ***
## s(Days):as.factor(Temp)30 2.617  2.617  2.768   0.0257 *
## ---
## Signif. codes:  0 '***' 0.001 '**' 0.01 '*' 0.05 '.' 0.1 ' ' 1
##
## R-sq.(adj) =  0.232
##   Scale est. = 36.257    n = 718
```

Fitted GAMM indicates significant differences between temperatures. The marginal tests for the significance of each individual smoothers are mostly highly significant indicating differences between the shape of the relaitonship between temperature and oviposition for each temperature.

```
temp.data <- expand.grid(Temp = c(20, 22, 24, 27, 30), Days = seq(2, 20, by = 0.1))

egg.predicted <- predict(egg.gam$gam, newdata = temp.data, se.fit = TRUE, type = "response")

rm(Days)
egg.predicted <- data.frame(temp.data, egg.predicted)

egg.predicted <- egg.predicted[-which(egg.predicted$Temp == 22 & egg.predicted$Days >
    18), ]
egg.predicted <- egg.predicted[-which(egg.predicted$Temp == 24 & egg.predicted$Days >
    16), ]
egg.predicted <- egg.predicted[-which(egg.predicted$Temp == 27 & egg.predicted$Days >
    12), ]
egg.predicted <- egg.predicted[-which(egg.predicted$Temp == 30 & egg.predicted$Days >
    10), ]

# attach(egg.predicted) plot(Days[Temp==20]-1,fit[Temp==20], type='l',
# ylim=c(0,100), col='darkblue', xlab='Day', ylab='Egg production per 48 hours')
# points(Days[Temp==20]-1,fit[Temp==20]+se.fit[Temp==20], type='l',
# ylim=c(0,100), col='darkblue',lty=3)
# points(Days[Temp==20]-1,fit[Temp==20]-se.fit[Temp==20], type='l',
# ylim=c(0,100), col='darkblue',lty=3) points(Days[Temp==22 & Days
# <=18]-1,fit[Temp==22& Days <=18], type='l', col='lightblue')
# points(Days[Temp==22 & Days <=18]-1,fit[Temp==22& Days <=18]+se.fit[Temp==22&
# Days <=18], type='l', col='lightblue', lty=3) points(Days[Temp==22 & Days
# <=18]-1,fit[Temp==22& Days <=18]-se.fit[Temp==22& Days <=18], type='l',
# col='lightblue', lty=3) points(Days[Temp==24 & Days <=16]-1,fit[Temp==24 & Days
# <=16], type='l', col='orange') points(Days[Temp==24 & Days <=16]-1,fit[Temp==24
# & Days <=16]+se.fit[Temp==24 & Days <=16], type='l', col='orange', lty=3)
# points(Days[Temp==24 & Days <=16]-1,fit[Temp==24 & Days <=16]-se.fit[Temp==24 &
# Days <=16], type='l', col='orange', lty=3) points(Days[Temp==27 & Days
# <=12]-1,fit[Temp==27 & Days <=12], type='l', col='red') points(Days[Temp==27 &
# Days <=12]-1,fit[Temp==27 & Days <=12]+se.fit[Temp==27 & Days <=12], type='l',
# col='red', lty=3) points(Days[Temp==27 & Days <=12]-1,fit[Temp==27 & Days
# <=12]-se.fit[Temp==27 & Days <=12], type='l', col='red', lty=3)
# points(Days[Temp==30 & Days <=10]-1,fit[Temp==30 & Days <=10], type='l',
# col='darkred') points(Days[Temp==30 & Days <=10]-1,fit[Temp==30 & Days
# <=10]+se.fit[Temp==30 & Days <=10], type='l', col='darkred', lty=3)
# points(Days[Temp==30 & Days <=10]-1,fit[Temp==30 & Days <=10]-se.fit[Temp==30 &
# Days <=10], type='l', col='darkred', lty=3) legend('topright',
# legend=c(expression(20 ~degree*C),expression(22 ~degree*C),expression(24
# ~degree*C),expression(27 ~degree*C),expression(30 ~degree*C)),
# col=c('darkblue','lightblue','orange','red','darkred'), lty=1, bty='n')
# detach(egg.predicted)

colnames(egglaying)[5] <- "Days_1"  #Need to have a different name for this otherwise ggplot gets upset


temp_labels <- c(`20` = "20°C", `22` = "22°C", `24` = "24°C", `27` = "27°C",
    `30` = "30°C")
```

```
egg.predicted$Temp <- factor(egg.predicted$Temp, levels = c("30", "27", "24", "22",
    "20"))

p1 <- ggplot(data = egg.predicted, aes(x = Days, y = fit)) + geom_line()
p1 <- p1 + geom_ribbon(aes(ymin = fit - se.fit, ymax = fit + se.fit), alpha = 0.3)
p1 <- p1 + geom_point(data = egglaying, aes(x = Days_1, y = Egg.count), colour = "dodgerblue3",
    fill = "dodgerblue3", stroke = 0, shape = 21, alpha = 0.5)
p1 <- p1 + facet_grid(. ~ Temp, labeller = labeller(Temp = temp_labels)) + theme_bw() +
    theme(panel.grid.major = element_blank(), panel.grid.minor = element_blank(),
        strip.background = element_rect(fill = "white", colour = "grey80"))
p1 <- p1 + xlab("Adult age (days)") + ylab("Egg production per 48 hours")

p1
```

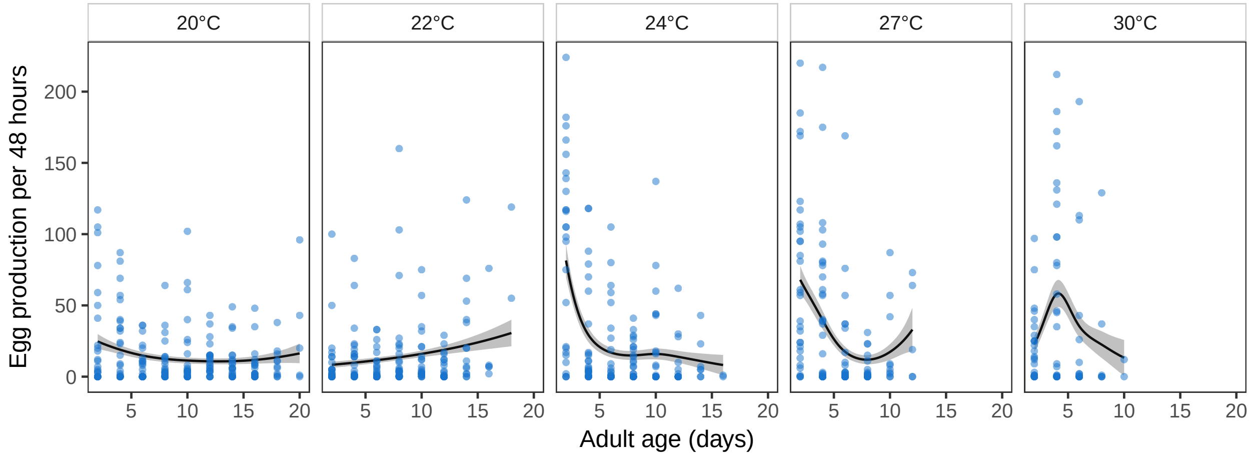


```
# ggsave('fig4.pdf',width = 15, height = 6, units = 'cm')

rm(list = ls())
```

Figure 4 Number of eggs laid per 48 hours plus the fitted smoothers from the GAMM for each temperature. At 20 degrees the moths live for a long time and produce a few eggs every day. The moths at 22 degrees have a similarly low oviposition rate but towards the end of their lives they greatly increase the number of eggs laid (NB some of the 20 degree moths did this as well but some did not). Once the temperature reaches 24 degrees and up oviposition is mostly early on in the animals' lives, with large numbers of eggs laid in the first few days.

6 Immunity measures and disease resistance

6.1 Haemocyte counts

6.1.1 Deletion test on initial model

```
################ Cell counts

cells <- read.csv("Laughton_et_al_haemocytes.csv")

Box <- as.numeric(as.factor(cells$Temperature))
```

```r
cells <- data.frame(cells, Box)
rm(Box)

cellmod1 <- lmer(sqrt(Mean.cell.count) ~ as.factor(Block) + I(1000 * Weight) + Temperature +
    (1 | Box), data = cells)

drop1(cellmod1, test = "Chisq")
```

```
## Single term deletions
##
## Model:
## sqrt(Mean.cell.count) ~ as.factor(Block) + I(1000 * Weight) +
##     Temperature + (1 | Box)
##                  Df    AIC     LRT   Pr(Chi)
## <none>              393.28
## as.factor(Block)  1 391.34  0.0551 0.8144171
## I(1000 * Weight)  1 391.73  0.4457 0.5043799
## Temperature       1 405.59 14.3123 0.0001549 ***
## ---
## Signif. codes:  0 '***' 0.001 '**' 0.01 '*' 0.05 '.' 0.1 ' ' 1
```

6.1.2 Deletion test on final model

```r
# simplify model

cellmod2 <- lmer(sqrt(Mean.cell.count) ~ Temperature + (1 | Box), data = cells)

drop1(cellmod2, test = "Chisq")
```

```
## Single term deletions
##
## Model:
## sqrt(Mean.cell.count) ~ Temperature + (1 | Box)
##             Df    AIC    LRT   Pr(Chi)
## <none>         389.79
## Temperature  1 401.94 14.151 0.0001687 ***
## ---
## Signif. codes:  0 '***' 0.001 '**' 0.01 '*' 0.05 '.' 0.1 ' ' 1
```

6.1.3 Summary of final model

```r
summary(cellmod2)
```

```
## Linear mixed model fit by REML t-tests use Satterthwaite approximations
##   to degrees of freedom [lmerMod]
## Formula: sqrt(Mean.cell.count) ~ Temperature + (1 | Box)
##    Data: cells
##
## REML criterion at convergence: 393.1
##
## Scaled residuals:
##      Min       1Q   Median       3Q      Max
```

```
## -2.39684 -0.66401 -0.06873  0.54810  2.91367
##
## Random effects:
##  Groups   Name        Variance Std.Dev.
##  Box      (Intercept) 0.000    0.0000
##  Residual             0.399    0.6316
## Number of obs: 200, groups:  Box, 5
##
## Fixed effects:
##              Estimate Std. Error        df t value Pr(>|t|)
## (Intercept)   1.36083    0.31134 198.00000   4.371 2.00e-05 ***
## Temperature   0.07342    0.01264 198.00000   5.808 2.49e-08 ***
## ---
## Signif. codes:  0 '***' 0.001 '**' 0.01 '*' 0.05 '.' 0.1 ' ' 1
##
## Correlation of Fixed Effects:
##             (Intr)
## Temperature -0.990
```

```
## Plot an equivalent barplot with error bars

attach(cells)

mean1 <- tapply(Mean.cell.count, Temperature, function(x) mean(sqrt(x)))
# sd1<-tapply(Slope, Temperature,sd) N1<-tapply(Slope, Temperature,length)
# se1<-sd1/sqrt(N1)
CI95 <- tapply(Mean.cell.count, Temperature, function(x) (sd(sqrt(x))/sqrt(length(x)) *
    qt(0.975, length(x) - 1)))

detach(cells)

# Predicted values from model
temp.data <- expand.grid(Temperature = seq(20, 30, length = 200), Box = 3)
test <- predict(cellmod2, newdata = temp.data, type = "response")

## Make data frame
temp <- c(20, 22, 24, 27, 30)
means <- data.frame(temp, mean1, CI95)
rm(temp)

## Plot figure

p2 <- ggplot(data = means, aes(x = temp, y = mean1))

p2 <- p2 + geom_bar(stat = "identity", colour = "black", fill = "grey60", size = 0.2)

p2 <- p2 + geom_errorbar(aes(ymin = mean1 - CI95, ymax = mean1 + CI95), width = 0.1,
    size = 0.2) + scale_x_continuous(breaks = c(20, 22, 24, 27, 30)) + ylim(0, 4)

p2 <- p2 + geom_line(data = temp.data, aes(x = Temperature, y = test), size = 0.2)

p2 <- p2 + theme_classic() + theme(panel.grid = element_blank()) + xlab(expression(Temperature ~
    degree * C)) + ylab("Mean square root haemocyte count") + scale_y_continuous(limits = c(0,
    4), expand = c(0, 0))
```

```
p2 <- p2 + theme(axis.line.y = element_line(colour = "black", size = 0.33), axis.line.x = element_line(colour = "blac
    size = 0.33))

p2
```

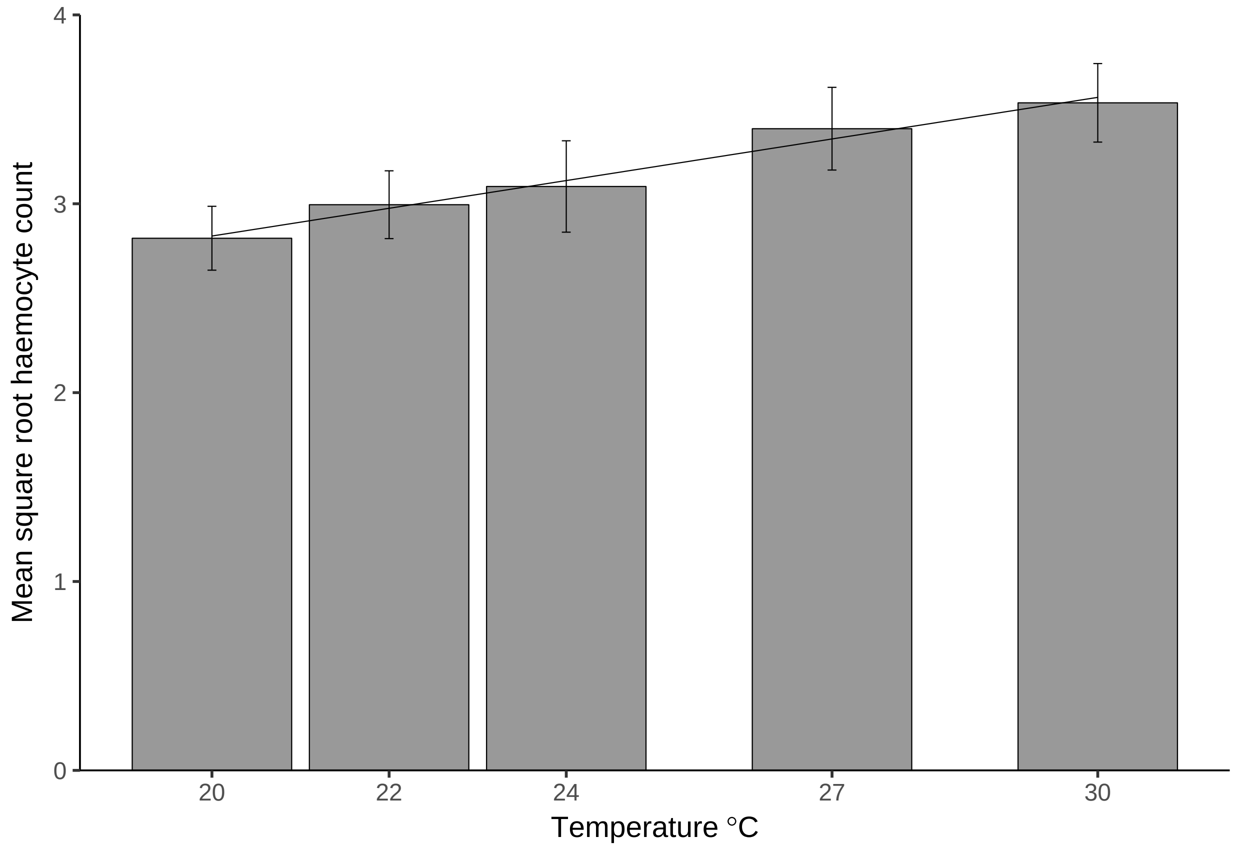


```
# ggsave('fig5.pdf', width = 12, height = 10, units = 'cm')

# rm(list=ls())
```

Figure 5 Means and 95% confidence intervals for square-root haemocyte count. The fitted line shows the predicted values from the model.

6.2 Phenoloxidase activity

6.2.1 Deletion test on initial model

```
######## PO
PO <- read.csv("Laughton_et_al_PO.csv")

Box <- as.numeric(as.factor(PO$Temperature))

PO <- data.frame(PO, Box)

rm(Box)

POmod1 <- lmer(sqrt(Slope) ~ Temperature + I(Temperature^2) + as.factor(Block) +
```

```r
    I(Weight * 1000) + (1 | Box), data = PO)

# NB weight expressed in mg rather than g to help with model fit

drop1(POmod1, test = "Chisq")

# simplify model
```

6.2.2 Deletion test on final model

```r
POmod2 <- lmer(sqrt(Slope) ~ Temperature + I(Temperature^2) + I(Weight * 1000) +
    (1 | Box), data = PO)

drop1(POmod2, test = "Chisq")
```

```
## Single term deletions
##
## Model:
## sqrt(Slope) ~ Temperature + I(Temperature^2) + I(Weight * 1000) +
##     (1 | Box)
##                  Df    AIC     LRT   Pr(Chi)
## <none>              420.29
## Temperature       1 424.79  6.4942 0.0108228 *
## I(Temperature^2)  1 424.18  5.8844 0.0152753 *
## I(Weight * 1000)  1 430.73 12.4346 0.0004215 ***
## ---
## Signif. codes:  0 '***' 0.001 '**' 0.01 '*' 0.05 '.' 0.1 ' ' 1
```

6.2.3 Summary of final model

```r
summary(POmod2)
```

```
## Linear mixed model fit by REML t-tests use Satterthwaite approximations
##   to degrees of freedom [lmerMod]
## Formula:
## sqrt(Slope) ~ Temperature + I(Temperature^2) + I(Weight * 1000) +
##     (1 | Box)
##    Data: PO
##
## REML criterion at convergence: 433.6
##
## Scaled residuals:
##     Min      1Q  Median      3Q     Max
## -2.8139 -0.6052  0.1527  0.6835  2.2057
##
## Random effects:
##  Groups   Name        Variance Std.Dev.
##  Box      (Intercept) 0.002642 0.0514
##  Residual             0.459076 0.6776
## Number of obs: 200, groups:  Box, 5
##
## Fixed effects:
```

```
##                      Estimate Std. Error         df t value Pr(>|t|)
## (Intercept)       -5.283355   3.353337   2.250000  -1.576 0.242036
## Temperature        0.694036   0.263770   1.990000   2.631 0.119744
## I(Temperature^2)  -0.012766   0.005256   1.990000  -2.429 0.136511
## I(Weight * 1000)  -0.093298   0.026326 195.970000  -3.544 0.000493 ***
## ---
## Signif. codes:  0 '***' 0.001 '**' 0.01 '*' 0.05 '.' 0.1 ' ' 1
##
## Correlation of Fixed Effects:
##              (Intr) Tmprtr I(T^2)
## Temperature -0.989
## I(Tmprtr^2)  0.983 -0.998
## I(Wgh*1000) -0.301  0.172 -0.161
```

```
## Plot as barplot with error bars

attach(PO)

mean1 <- tapply(Slope, Temperature, function(x) sqrt(mean(x)))
sd1 <- tapply(Slope, Temperature, sd)
N1 <- tapply(Slope, Temperature, length)
se1 <- sd1/sqrt(N1)
CI95 <- tapply(Slope, Temperature, function(x) (sd(sqrt(x))/sqrt(length(x))) * qt(0.975,
    length(x) - 1))

detach(PO)

## Make data frame
temp <- c(20, 22, 24, 27, 30)
means <- data.frame(temp, mean1, sd1, se1, CI95)
rm(temp)

### Extract coefficients from model
X1 <- coefficients(POmod2)

POmod_intercept <- mean(X1$Box[, 1])
POmod_slope <- mean(X1$Box[, 2])
POmod_quadratic <- mean(X1$Box[, 3])

### Generate predicted values

temp.data <- expand.grid(Temperature = seq(20, 30, length = 200), Weight = median(PO$Weight),
    Box = 3)
test <- predict(POmod2, newdata = temp.data, type = "response")



### Plot data
p2 <- ggplot(data = means, aes(x = temp, y = mean1))


### Use this instead of the next line for points with error bars p2<- p2+
### geom_errorbar(aes(ymin=mean1-se1, ymax=mean1+se1), width=.1) +
### geom_point(size=3, shape=21,
```

```
### fill='white')+scale_x_continuous(breaks=c(20,22,24,27,30))



p2 <- p2 + geom_bar(stat = "identity", colour = "black", fill = "grey60", size = 0.2)

p2 <- p2 + geom_errorbar(aes(ymin = mean1 - CI95, ymax = mean1 + CI95), width = 0.1,
    size = 0.2) + scale_x_continuous(breaks = c(20, 22, 24, 27, 30)) + ylim(0, 3)

p2 <- p2 + geom_line(data = temp.data, aes(x = Temperature, y = test), size = 0.2)

# p2<-p2+stat_function(fun=function(x) POmod_intercept + POmod_slope*x +
# POmod_quadratic[3]*(x^2), size=0.2) ## What's going on here - how did you get
# the coordinates for the curve?
p2 <- p2 + theme_classic() + theme(panel.grid = element_blank()) + xlab(expression(Temperature ~
    degree * C)) + ylab("PO activity (square root of maximum slope)") + scale_y_continuous(limits = c(0,
    3), expand = c(0, 0))
p2 <- p2 + theme(axis.line.y = element_line(colour = "black", size = 0.33), axis.line.x = element_line(colour = "blac
    size = 0.33))

p2
```

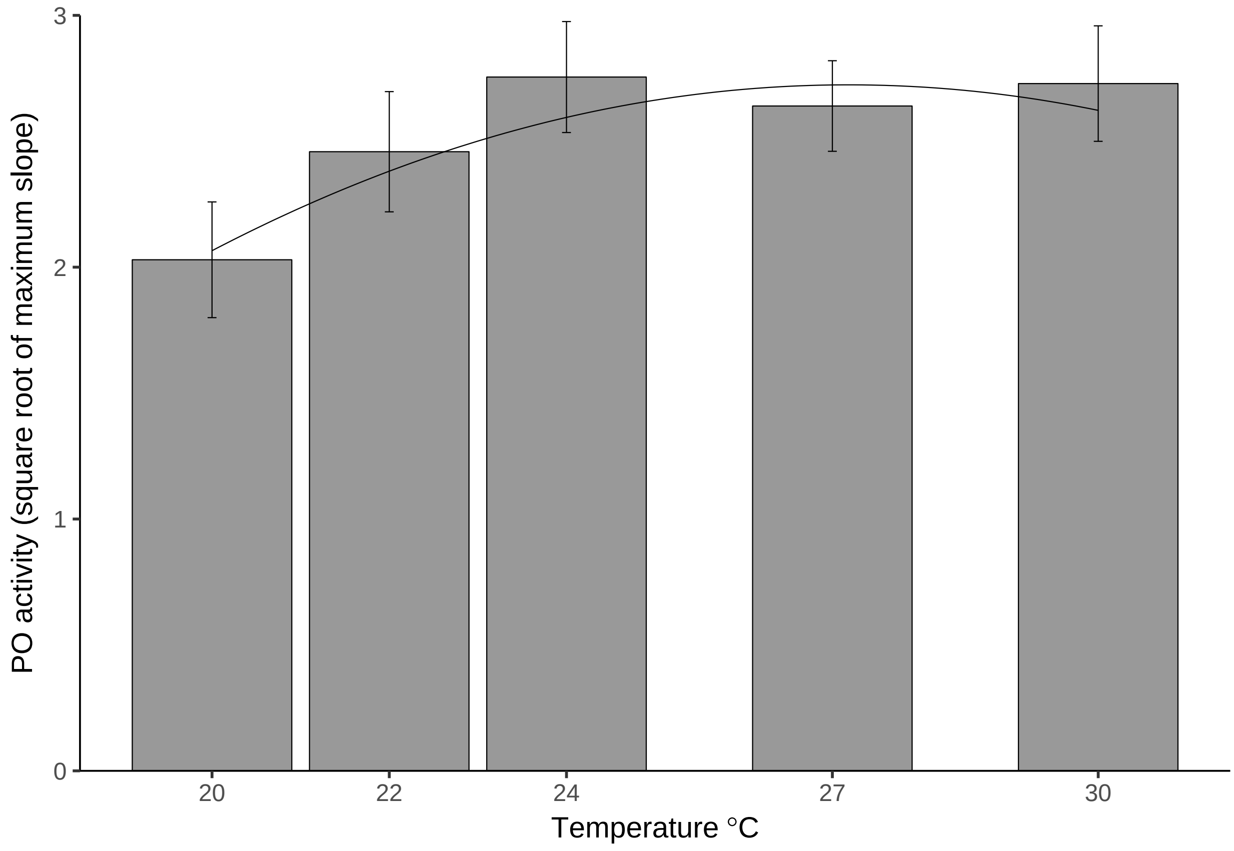


```
# ggsave('fig6.pdf', width= 12, height = 10, units = 'cm')
```

Figure 6 Phenoloxidase activity plotted against temperature. The line indicates predicted values from the fitted model with the median value for median larval weight and the third incubator. Error bars are 95% confidence intervals.

6.3 Correlation between haemocyte count and PO activity

```r
PO_Haem_mod1 <- lmer(sqrt(PO) ~ Temperature + I(Temperature^2) + I(Weight * 1000) +
    Mean.cell.count + (1 | Box), data = cells)

drop1(PO_Haem_mod1)
```

```
## Single term deletions
##
## Model:
## sqrt(PO) ~ Temperature + I(Temperature^2) + I(Weight * 1000) +
##     Mean.cell.count + (1 | Box)
##                   Df    AIC
## <none>               412.32
## Temperature       1 416.27
## I(Temperature^2)  1 415.87
## I(Weight * 1000)  1 424.42
## Mean.cell.count   1 420.29
```

```r
summary(PO_Haem_mod1)
```

```
## Linear mixed model fit by REML t-tests use Satterthwaite approximations
##   to degrees of freedom [lmerMod]
## Formula: sqrt(PO) ~ Temperature + I(Temperature^2) + I(Weight * 1000) +
##     Mean.cell.count + (1 | Box)
##    Data: cells
##
## REML criterion at convergence: 430.9
##
## Scaled residuals:
##     Min      1Q  Median      3Q     Max
## -2.9418 -0.5927  0.1133  0.6532  2.5530
##
## Random effects:
##  Groups   Name        Variance Std.Dev.
##  Box      (Intercept) 0.005324 0.07297
##  Residual             0.437867 0.66171
## Number of obs: 200, groups:  Box, 5
##
## Fixed effects:
##                    Estimate Std. Error         df t value Pr(>|t|)
## (Intercept)       -4.860920   3.574056   2.210000  -1.360 0.295812
## Temperature        0.651638   0.282662   2.000000   2.305 0.147669
## I(Temperature^2)  -0.012268   0.005629   1.990000  -2.179 0.161622
## I(Weight * 1000)  -0.097200   0.025765 194.980000  -3.773 0.000214 ***
## Mean.cell.count    0.035681   0.011210 193.200000   3.183 0.001700 **
## ---
## Signif. codes:  0 '***' 0.001 '**' 0.01 '*' 0.05 '.' 0.1 ' ' 1
##
## Correlation of Fixed Effects:
##             (Intr) Tmprtr I(T^2) I(W*10
## Temperature -0.991
## I(Tmprtr^2)  0.985 -0.998
## I(Wgh*1000) -0.277  0.159 -0.148
## Men.cll.cnt  0.035 -0.045  0.026 -0.048
```

```
palette2 <- c("Purple", "Blue", "Green4", "darkorange", "firebrick3")

p1 <- ggplot(data = cells, aes(x = sqrt(Mean.cell.count), y = sqrt(PO))) + geom_point(colour = "steelblue",
    fill = "steelblue", stroke = 0, alpha = 0.9)
# p1 <- ggplot(data = cells, aes(x = Mean.cell.count, y = sqrt(PO)) +
# geom_point() + theme_classic()
p1 <- p1 + geom_smooth(method = "lm", colour = "grey10", size = 0.2) + theme_classic()
p1 <- p1 + xlab("Mean square root haemocyte count") + ylab("Square root Phenoloxidase activity")
p1
```

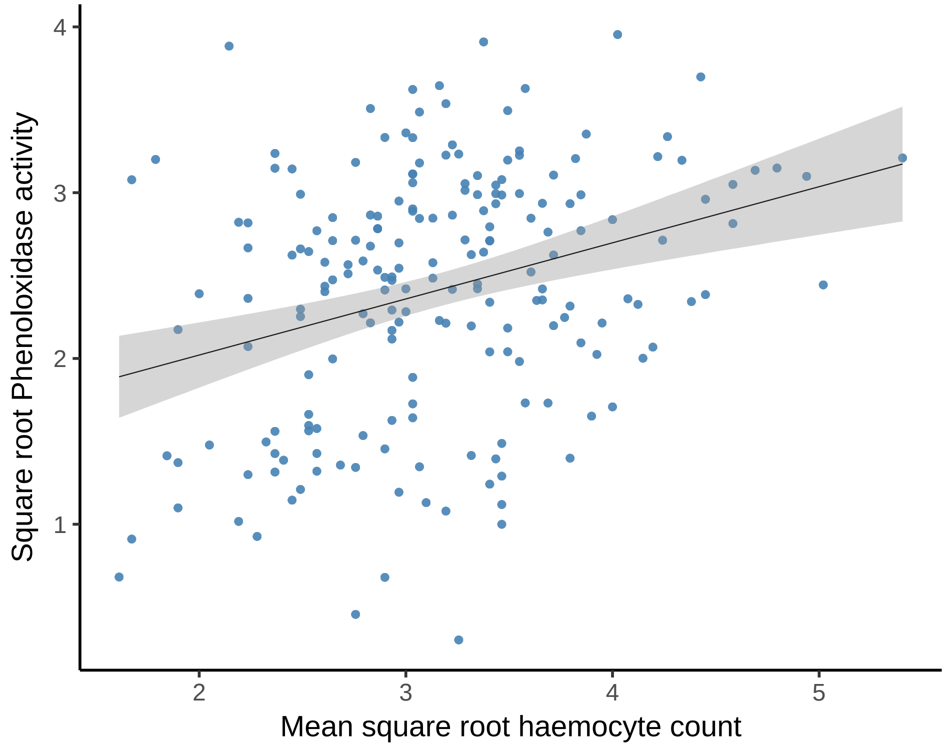


```
# ggsave('fig7.pdf', width = 12, height = 10, units = 'cm')
```

Figure 7 Square root PO activity plotted against square root haemocyte count. The line is for illustrative purposes only and shows the results of a simple linear regression through the data.

6.4 Bacterial clearance

```
####### Bacteria

bacteria <- read.csv("Laughton_et_al_bacterial_clearance.csv")

Box <- as.numeric(as.factor(bacteria$Temperature))

bacteria <- data.frame(bacteria, Box)

rm(Box)

Bac.mod <- lmer((log(Total.bacteria)) ~ Temperature + I(Temperature^2) + I(Temperature^3) +
    as.factor(Block) + I(1000 * Weight) + (1 | Box), data = bacteria)
```

```r
drop1(Bac.mod, test = "Chisq")
```

```
## Single term deletions
##
## Model:
## (log(Total.bacteria)) ~ Temperature + I(Temperature^2) + I(Temperature^3) +
##     as.factor(Block) + I(1000 * Weight) + (1 | Box)
##                  Df    AIC    LRT  Pr(Chi)
## <none>              1038.8
## Temperature       1 1046.2 9.4323 0.002132 **
## I(Temperature^2)  1 1046.1 9.3014 0.002290 **
## I(Temperature^3)  1 1046.0 9.1645 0.002468 **
## as.factor(Block)  1 1037.4 0.6125 0.433834
## I(1000 * Weight)  1 1037.1 0.3398 0.559930
## ---
## Signif. codes:  0 '***' 0.001 '**' 0.01 '*' 0.05 '.' 0.1 ' ' 1
```

```r
# simplify model

Bac.mod2 <- lmer((log(Total.bacteria)) ~ Temperature + I(Temperature^2) + I(Temperature^3) +
    (1 | Box), data = bacteria)
```

6.4.1 Deletion test on final model

```r
drop1(Bac.mod2, test = "Chisq")
```

```
## Single term deletions
##
## Model:
## (log(Total.bacteria)) ~ Temperature + I(Temperature^2) + I(Temperature^3) +
##     (1 | Box)
##                  Df    AIC    LRT  Pr(Chi)
## <none>              1035.7
## Temperature       1 1043.1 9.4401 0.002123 **
## I(Temperature^2)  1 1043.0 9.3097 0.002279 **
## I(Temperature^3)  1 1042.8 9.1750 0.002453 **
## ---
## Signif. codes:  0 '***' 0.001 '**' 0.01 '*' 0.05 '.' 0.1 ' ' 1
```

6.4.2 Summary of final model

```r
summary(Bac.mod2)
```

```
## Linear mixed model fit by REML t-tests use Satterthwaite approximations
##   to degrees of freedom [lmerMod]
## Formula:
## (log(Total.bacteria)) ~ Temperature + I(Temperature^2) + I(Temperature^3) +
##     (1 | Box)
##    Data: bacteria
##
## REML criterion at convergence: 1048.1
##
```

```
## Scaled residuals:
##     Min      1Q  Median      3Q     Max
## -2.7379 -0.6246 -0.1118  0.5917  2.7280
##
## Random effects:
##  Groups   Name        Variance  Std.Dev.
##  Box      (Intercept) 1.719e-13 4.146e-07
##  Residual             2.914e+00 1.707e+00
## Number of obs: 263, groups:  Box, 5
##
## Fixed effects:
##                     Estimate Std. Error         df t value Pr(>|t|)
## (Intercept)       219.333760  57.782553 259.000000   3.796 0.000183 ***
## Temperature       -25.780808   7.071318 259.000000  -3.646 0.000322 ***
## I(Temperature^2)    1.027663   0.285571 259.000000   3.599 0.000383 ***
## I(Temperature^3)   -0.013512   0.003807 259.000000  -3.549 0.000458 ***
## ---
## Signif. codes:  0 '***' 0.001 '**' 0.01 '*' 0.05 '.' 0.1 ' ' 1
##
## Correlation of Fixed Effects:
##             (Intr) Tmprtr I(T^2)
## Temperature -0.999
## I(Tmprtr^2)  0.997 -0.999
## I(Tmprtr^3) -0.994  0.997 -0.999
## fit warnings:
## Some predictor variables are on very different scales: consider rescaling
```

```
attach(bacteria)

mean1 <- tapply(Total.bacteria, Temperature, function(x) mean(log(x)))
# sd1<-tapply(Slope, Temperature,sd) N1<-tapply(Slope, Temperature,length)
# se1<-sd1/sqrt(N1)
CI95 <- tapply(Total.bacteria, Temperature, function(x) (sd(log(x))/sqrt(length(x)) *
    qt(0.975, length(x) - 1)))

detach(bacteria)

# Predicted values from model
temp.data <- expand.grid(Temperature = seq(20, 30, length = 200), Box = 3)
test <- predict(Bac.mod2, newdata = temp.data, type = "response")

## Make data frame
temp <- c(20, 22, 24, 27, 30)
means <- data.frame(temp, mean1, CI95)
rm(temp)

## Plot figure

p2 <- ggplot(data = means, aes(x = temp, y = mean1))

p2 <- p2 + geom_bar(stat = "identity", colour = "black", fill = "grey60", size = 0.2)

p2 <- p2 + geom_errorbar(aes(ymin = mean1 - CI95, ymax = mean1 + CI95), width = 0.1,
    size = 0.2) + scale_x_continuous(breaks = c(20, 22, 24, 27, 30)) + ylim(0, 7.5)
```

```r
p2 <- p2 + geom_line(data = temp.data, aes(x = Temperature, y = test), size = 0.33)

p2 <- p2 + theme_classic() + theme(panel.grid = element_blank()) + xlab(expression(Temperature ~
    degree * C)) + ylab("Mean log bacterial count") + scale_y_continuous(limits = c(0,
    7.5), expand = c(0, 0))
p2 <- p2 + theme(axis.line.y = element_line(colour = "black", size = 0.33), axis.line.x = element_line(colour = "blac
    size = 0.2))

p2
```

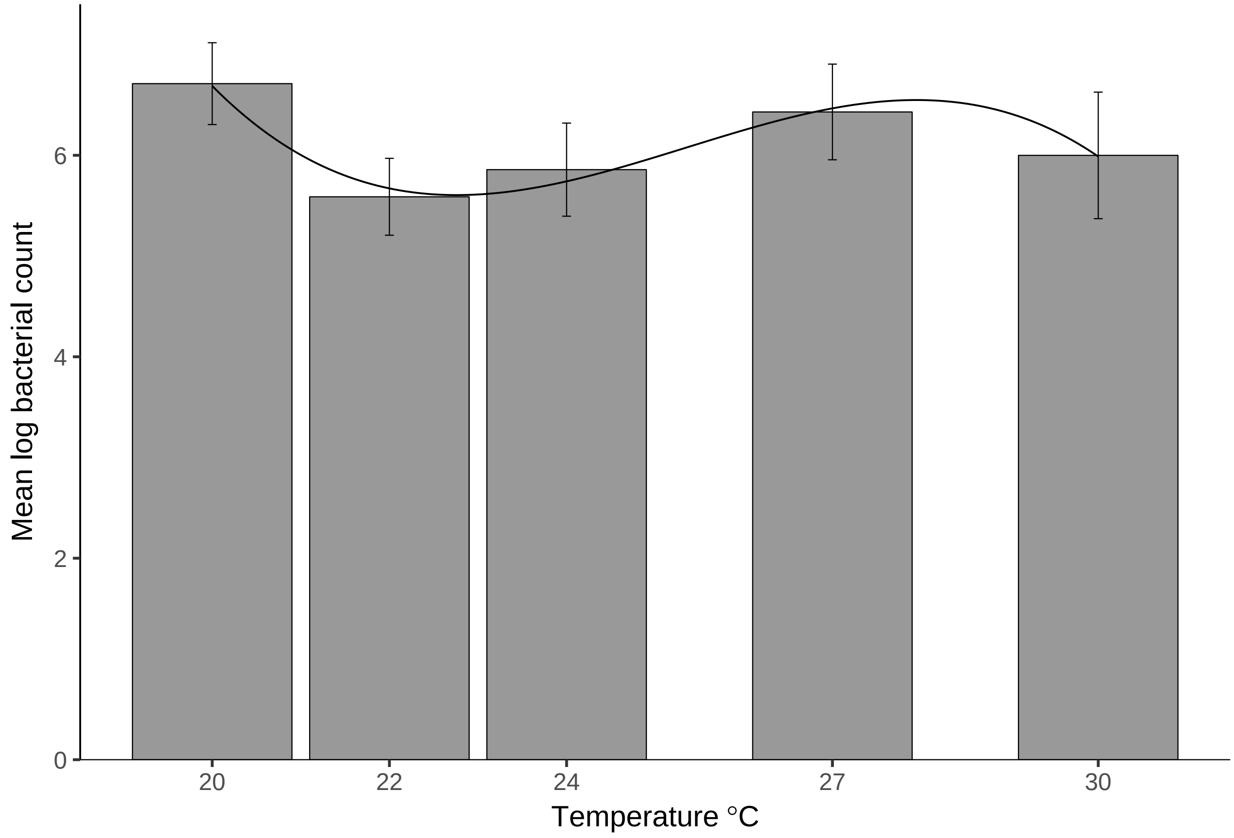


```r
# ggsave('fig8.pdf', width = 12, height = 10, units = 'cm')

# rm(list=ls())
```

Figure 8 Means and 95% confidence intervals for log bacterial counts. The fitted line shows the predicted values from the model.

6.5 Virus resistance

6.5.1 Deletion test on model

```r
############## Virus

virus <- read.csv("Laughton_et_al_virus_resistance.csv")

Box <- as.numeric(as.factor(virus$End.temp))
```

```r
virus <- data.frame(virus, Box)
rm(Box)

virusmod1 <- glmer(cbind(Infected, Uninfected) ~ Start * End.temp + (1 | Box), family = binomial,
    data = virus)

drop1(virusmod1, test = "Chisq")
```

```
## Single term deletions
##
## Model:
## cbind(Infected, Uninfected) ~ Start * End.temp + (1 | Box)
##                Df    AIC    LRT   Pr(Chi)
## <none>            64.280
## Start:End.temp  1 76.275 13.995 0.0001833 ***
## ---
## Signif. codes:  0 '***' 0.001 '**' 0.01 '*' 0.05 '.' 0.1 ' ' 1
```

6.5.2 Summary of final model

```r
summary(virusmod1)
```

```
## Generalized linear mixed model fit by maximum likelihood (Laplace
##   Approximation) [glmerMod]
##  Family: binomial  ( logit )
## Formula: cbind(Infected, Uninfected) ~ Start * End.temp + (1 | Box)
##    Data: virus
##
##      AIC      BIC   logLik deviance df.resid
##     64.3     65.8    -27.1     54.3        5
##
## Scaled residuals:
##     Min      1Q  Median      3Q     Max
## -1.4361 -1.0312 -0.0126  0.5938  1.6700
##
## Random effects:
##  Groups Name        Variance Std.Dev.
##  Box    (Intercept) 0.1444   0.38
## Number of obs: 10, groups:  Box, 5
##
## Fixed effects:
##                              Estimate Std. Error z value Pr(>|z|)
## (Intercept)                   6.09607    1.93614   3.149 0.001641 **
## Startstandard lab            -8.16244    2.05449  -3.973  7.1e-05 ***
## End.temp                     -0.22836    0.07761  -2.942 0.003257 **
## Startstandard lab:End.temp    0.29740    0.08200   3.627 0.000287 ***
## ---
## Signif. codes:  0 '***' 0.001 '**' 0.01 '*' 0.05 '.' 0.1 ' ' 1
##
## Correlation of Fixed Effects:
##             (Intr) Strtsl End.tm
## Strtstndrdl -0.591
## End.temp    -0.991  0.586
```

```
## Strtlb:End.  0.588 -0.991 -0.592
```

The significant interaction term indicates that the relationship between virus resistance and temperature depends on the temperature experienced prior to infection. When animals were kept at 26 degrees prior to virus exposure there is no real effect of post-exposure temperature, but when animals were kept at the treatment temperature before exposure as well as after it virus resistance increases with temperature.

```
Lower95 <- numeric(10)
Upper95 <- numeric(10)

for (i in 1:10) {
    # Calculate binomial confidence intervals
    temp <- binom.test(virus$Infected[i], virus$Total.sample[i])
    Lower95[i] <- temp$conf.int[1]
    Upper95[i] <- temp$conf.int[2]

}

## Generate predicted values

temp.data <- expand.grid(End.temp = seq(20, 30, length = 200), Start = c("standard lab",
    "continuous"), Box = 3)
test <- predict(virusmod1, newdata = temp.data, type = "response")
temp.data <- data.frame(temp.data, test)


data <- data.frame(virus, Lower95, Upper95)

rm(Lower95, Upper95)

attach(data)



dodge <- position_dodge(width = 0.5)  #Need to specify dodge width because bars and errorbars are not the same width

p1 <- ggplot(data = data, aes(x = End.temp, y = percent, colour = Start))  #Set up basics - x,y,etc.

p1 <- p1 + geom_errorbar(aes(ymin = Lower95, ymax = Upper95), position = dodge, width = 0.2,
    size = 0.3)  #draw errorbars in.


p1 <- p1 + geom_point(aes(shape = Start), position = dodge, size = 3)

p1 <- p1 + scale_x_continuous(breaks = c(20, 22, 24, 27, 30)) + scale_y_continuous(limits = c(0,
    1), expand = c(0, 0))  #Set geom to bar plot, position_dodge makes them side by side, scale_x_continuous sets the


p1 <- p1 + geom_line(data = temp.data, aes(x = End.temp, y = test, colour = Start),
    size = 0.33)

p1 <- p1 + theme_classic() + theme(panel.grid = element_blank()) + xlab(expression(Temperature ~
    degree * C)) + ylab("Percent mortality from PiGV")  #Get rid of all the usual ggplot2 shit - grey background, gri

p1 <- p1 + scale_colour_manual(values = c("darkblue", "firebrick3"), name = "Treatment",
```

```
    labels = c("Constant pre-and post-infection temp.", "26C pre-infection temp.")) +
    theme(legend.position = c(0.7, 0.92), legend.title = element_blank(), axis.line.y = element_line(colour = "black"
        size = 0.33), axis.line.x = element_line(colour = "black", size = 0.33))  #Make the legend say what it should

p1 <- p1 + scale_shape_manual(values = c(19, 17), name = "Treatment", labels = c("Constant pre-and post-infection tem
    "26C pre-infection temp.")) + theme(legend.position = c(0.7, 0.92), legend.title = element_blank(),
    axis.line.y = element_line(colour = "black", size = 0.33), axis.line.x = element_line(colour = "black",
        size = 0.33))  #Make the legend say what it should say and move it to within the plot area


p1
```

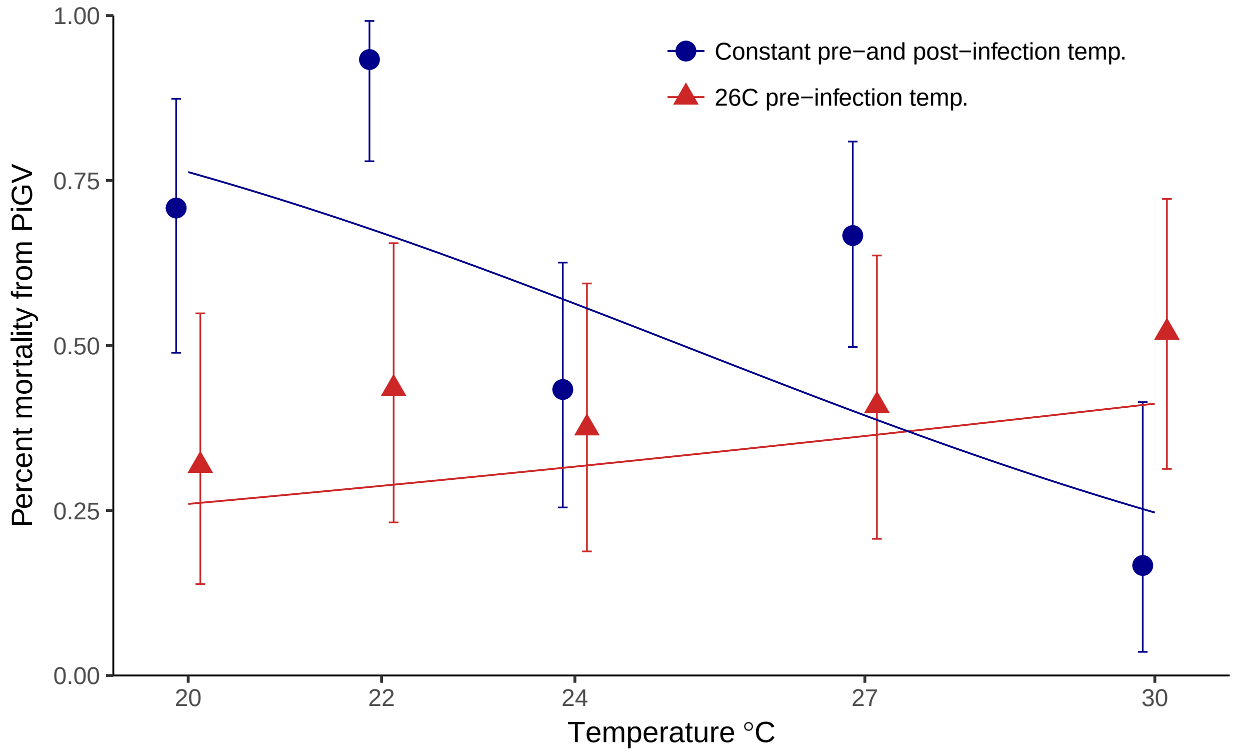


```
# ggsave('fig9.pdf', width = 12, height = 10, units = 'cm')

detach(data)
rm = list(ls)
```

Figure 9 Virus resistance against post-exposure temperature. Lines indicate predicted values from the fitted model.
